# High-Throughput Targeted Paleoproteomics Sex Estimation on Medieval Great Moravian Individuals Using MALDI-CASI-FTICR Mass Spectrometry

**DOI:** 10.1021/acs.jproteome.6c00163

**Published:** 2026-08-28

**Authors:** Fabrice Bray, Anežka Pilmann Kotěrová, Lisa Garbé, Marc Haegelin, Benoît Bertrand, Kevimy Agossa, Christian Rolando, Petr Velemínský, Jaroslav Brůžek, Marine Morvan

**Affiliations:** † 27023 Univ. Lille, CNRS, UAR 2638 - IMEC − Institut Michel Eugène Chevreul, Lille F-59000, France; ‡ Department of Anthropology and Human Genetics, Faculty of Science, Charles University, Viničná 7, Prague 2 128 00, Czech Republic; § Univ. Lille, CHU Lille, ULR 7367 - UTML&A - Unité de Taphonomie Médico-Légale & d’Anatomie, Lille F-59000, France; ∥ 27023 Univ. Lille, CHU Lille, School of Dentistry, Department of Periodontology, Lille 59000, France; ⊥ Department of Anthropology, National Museum, Václavské náměstí 1700/68, Prague 1 110 00, Czech Republic; # 54694 Vilnius University, Faculty of History, Department of Archaeology, Universiteto g. 7, Vilnius LT-01513, Lithuania

**Keywords:** amelogenin, biological sex, high-throughput, MALDI-FTICR, MALDI-CASI-FTICR, mass spectrometry, proteomics, tooth enamel

## Abstract

The estimation of the biological sex of archeological remains is crucial information in bioarcheology and forensic anthropology. In recent years, proteomics based on molecular sexual dimorphism has emerged as a preferred method, particularly because of its minimally invasive approach for extracting amelogenin X and Y proteins from tooth enamel. However, there is an increasing demand to accelerate this process and facilitate the analysis of large archeological assemblages. This study presents a novel high-throughput targeted paleoproteomics method for biological sex estimation using MALDI-CASI-FTICR mass spectrometry. This approach combines the strengths of existing methods, including ultrahigh resolution, significantly reduced processing times, targeted analysis, and scalability to large archeological sample sets. The method was initially validated on modern individuals with known sex and subsequently applied to 130 adult and juvenile individuals from medieval Great Moravia (present-day Czech Republic). Biological sex was successfully estimated for all but one of the individuals. The results not only provide a more efficient biological sex estimation but also help to resolve a few errors in sex assessment previously encountered with osteomorphological and tooth morphometric techniques. The implementation of this method significantly improves the accuracy and efficiency of biological sex estimation, offering a powerful tool for anthropological research.

## Graphical abstract

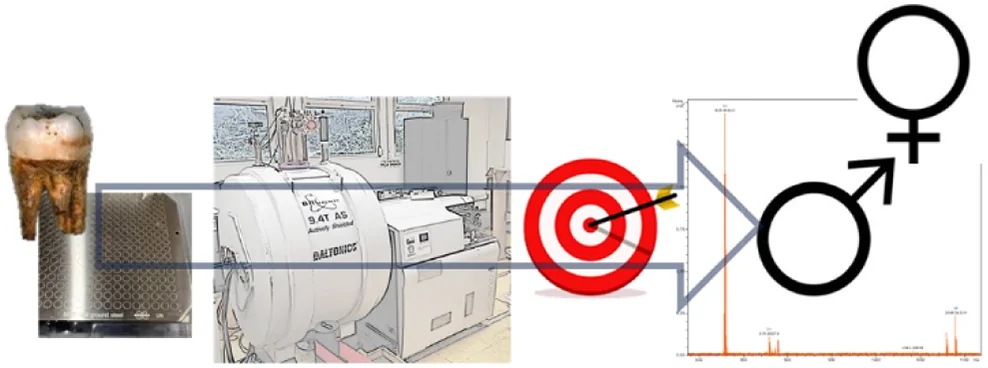

## Introduction

The estimation of the biological sex of human skeletal remains is a core component of both bioarcheological inference and forensic identification of human remains. Sex estimation provides a structural basis for developing other key elements of the biological profile. Morphological sex assessment is not fully independent, and the validity of morphological assessment is enhanced by the reliable estimation of other biological parameters, particularly age-at-death and ancestry/population affinity. It is well established that the reliability of skeletal sex assessment is sensitive to multiple sources of variation and bias, including inter- and intrapopulation differences, ontogenetic effects, pathological conditions or taphonomic alterations that may obscure morphological sex assessment. In particular, it is generally inadvisible to attempt morphological sex assessment in fetal, infant, and juvenile remains, as valid and sufficiently accurate osteological methods are not available owing to the limited expression of sexual dimorphism before puberty.^1^ In cases where skeletal sex assessment is not feasible or is expected to be unreliable, such as highly fragmentary assemblages or subadult remains, molecular or biochemical methods may provide a useful alternative.

Proteomics approaches based on molecular sexual dimorphism have emerged as powerful tools in recent years,^2^ particularly through the minimally invasive recovery of sex-linked amelogenin (AMELX/AMELY) peptides from tooth enamel,^3^ applicable to both adult and subadult individuals.^1,4^ Owing to its limited invasiveness and the superior preservation of enamel proteins relative to DNA,^2^ proteomics analysis has become a preferred method for biological sex estimation. Amelogenin, a sex-specific protein present in tooth enamel, occurs as two isoforms—AMELX and AMELY—encoded by the X and Y chromosomes, respectively. Using mass spectrometry, peptides derived from these isoforms can be reliably distinguished, enabling accurate estimation of biological sex.^5^ This approach has also proven effective in resolving sex attribution errors inherent to osteomorphological assessments.^3,6^

Currently, there is strong pressure to accelerate the entire process of biological sex estimation while simultaneously enabling the analysis of a large number of samples per day.^7–11^ Recent Liquid Chromatography-High Resolution Mass Spectrometry (LC-HRMS) studies have demonstrated that modern workflows based on minimally destructive enamel peptide extraction via acid etching combined with targeted proteomics allow for high-throughput (up to 200 samples per day) with high quantitative accuracy and reliable sex estimation, even in archeological material.^7,8^ Another targeted LC-HRMS study showed the possibility of analyzing up to 480 samples per day, but without the use of commercial standards.^12^ These methods reduce total analysis time to a few minutes up to tens of minutes per sample without compromising the number of identified peptides or the quality of biological sex estimation. These approaches pave the way for the analysis of entire populations rather than focusing on individual well-preserved specimens.

Both untargeted^3,13,14^ and targeted^7,12,15,16^ approaches have recently been used for biological sex estimation. The untargeted approach allows an exploratory study due to the analysis of the amelogenin sex-dependent peptides, and all other proteins and peptides present in the sample, including contaminations, which, in the case of archeological samples, can represent a significant ratio. Conversely, the targeted approach allows only preselected amelogenin sex-dependent peptides to be analyzed, regardless of contamination degree. This approach offers the possibility of quantifying peptides of interest for improved biological sex estimation.

The use of commercial standard peptides strategy allows for the most confident sex estimation. Two different approaches have recently been used. The first, external calibration, used by Koenig et al.,^7^ involves measuring synthetic peptide standards with known concentrations to generate a calibration curve, which is then used to identify the targeted peptides in the analyzed samples. This approach is simple and effective for purified matrices but is sensitive to instrumental variations during the successive analyses of large cohorts. The second method, internal calibration, used by Froment et al.,^16^ involves measuring synthetic isotope-labeled peptide standards with known concentrations to generate a calibration curve. A known and controlled concentration of isotope-labeled synthetic peptide standards is also added to each sample and can be distinguished by mass spectrometry through their mass shift due to isotopic enrichment. The biological sex estimation was based on the ratio of the intensity of the targeted peptide (light, into the sample) to that of the internal isotope-labeled peptide (heavy, controlled addition into the sample). This approach provides higher accuracy and reliability, can be applied to unpurified matrices, and is not sensitive to instrumental variations; however, the preparation requires an additional step than external calibration.

Matrix-Assisted Laser Desorption/Ionization Mass Spectrometry (MALDI-MS), for its part, is rarely used for the estimation of biological sex. Two studies reported the use of Matrix-Assisted Laser Desorption/Ionization-Time-of-Flight (MALDI-TOF)^17,18^ or, more recently, Liquid Atmospheric Pressure-Matrix-Assisted Laser Desorption/Ionization-Time-of-Flight (LAP-MALDI-TOF)^9,10^ for untargeted biological sex estimation of human teeth remains.

Here, we present the first study using a high-throughput targeted Matrix-Assisted Laser Desorption/Ionization coupled with Continuous Accumulation of Selected Ions and Fourier Transform Ion Cyclotron Resonance Mass Spectrometry (MALDI-CASI-FTICR MS) method for biological sex estimation of archeological remains with internal standards. This method combines the advantages of all methods commonly used to date, including extremely high resolution, significantly shorter processing time, high suitability for highly degraded material, and insensitivity to instrumental variations. These advantages enable the rapid and efficient screening of large sample sets. The targeted MALDI-CASI-FTICR MS workflow further enhances the analytical sensitivity and selectivity, facilitating reliable internal identification of the sex-specific amelogenin peptides AMELX and AMELY. Together, these features establish a rapid, high-throughput, and analytically robust alternative for sex estimation in proteomics, particularly well-suited for large archeological assemblages and minimally destructive sampling strategies. The method was first designed for modern individuals whose sex is known and was subsequently applied to archeological teeth from two Early Medieval Great Moravian burial sites in the present-day Czech Republic, comprising both adult and juvenile individuals. In this study, we chose the terminology *biological sex estimation* when relating to biological techniques such as proteomics and aDNA, and *sex assessment* when relating to anthropology.

## Materials and Methods

### Samples and Osteological Analysis

Permanent modern teeth (n = 28; 14 males, 14 females) from adult individuals of known sex, extracted for therapeutic indications in routine dental practice were obtained for assay development. Deciduous modern teeth (n= 8; 4 males, 4 females) from juvenile individuals of known sex, naturally exfoliated during childhood, were collected for assay development. Use for research was based on written informed consent within an approved ethical framework, with voluntary participation, nonimpacting clinical care, and samples handled under confidentiality safeguards. Specimens were provided either via the Forensic Taphonomy and Anatomy Unit or through the Faculty of Odontology (University of Lille, France).

The archeological samples were obtained from two archeologically significant Early Medieval Great Moravian burial sites (present-day Czech Republic). First, 30 adult teeth originating from the excavation of the third church at the medieval site of Mikulčice (9th–10th centuries) in South Moravia were used in the study. The sex and age-at-death of the adult individuals were previously assessed by Stloukal.^19,20^ A revised assessment of sex and age was later published by Zazvonilová et al.^21^ using various osteomorphological methods. Sex of some individuals was also assessed by Brůžek and Velemínský in 2017 (unpublished) based on primary and secondary sex diagnosis^22^ (Table 2).

Second, 100 juvenile teeth (both deciduous and permanent) from another Early Medieval Great Moravian necropolis located in Rajhrad (9th–10th centuries) were analyzed. Osteobiographical analysis was carried out by Hanáková et al.;^23^ however, sex assessment was not performed for nonadult individuals. In addition, the sex of 13 individuals was previously assessed by Pospíšilová^24^ using the permanent teeth metric method^25^ (Table 3).

### Reagents and Chemicals

All aqueous solutions were prepared using ultrapure grade water obtained by water filtration with a Direct-Q3 system (Merck Millipore, Burlington, Massachusetts, United States). All chemicals, biochemicals, and solvents were purchased from Merck (Merck KGaA, Darmstadt, Germany) and used without additional purification. All solvents were of MS analytical grade (Thermo Fisher, Kandel, Germany).

### Peptide Extraction by Acid Etching and Purification

Each tooth was individually washed with LC-MS-grade water. The resulting wash solution was subsequently analyzed using the same analytical procedure to evaluate potential contamination. The tooth was then incubated in 200 μL of 0.6 N HCl at room temperature for 1 h. The crown of the tooth was incubated in the cap of 1.5 mL microcentrifuge tube. Enamel peptide solutions were then purified using 96-well plates C18 (Affinisep, Petit-Couronne, France). The peptides were resuspended in 10 μL of 0.1% formic acid in H_2_O. An extraction blank was processed in parallel with the samples following the same procedure to identify any potential contaminants. In addition, a purification blank, processed through all C18 purification steps in an empty well on the same plate as the samples, was included to assess potential well-to-well cross-contamination.

### Untargeted and Targeted MALDI-FTICR MS Analysis

The desalted peptides were diluted 2 times in 0.1% formic acid in H_2_O. Diluted peptides (0.6 μL) were deposited on 384 Ground steel MALDI plates (Bruker Daltonics, Bremen, Germany) with 0.6 μL of isotope-labeled peptides (AQUA Peptides, Merck KGaA) at 6 fmol for AMELX and AMELY (Table 1) and 1 μL of CHCA matrix at 10 mg/mL in ACN/H_2_O (80/20, *v/v*). TFA (0.1%) was added to each sample spot and dried at ambient temperature.

**Table 1:** List of Isotope-Labeled Peptides for AMELX and AMELY (AQUA Peptides)^a^

Peptide sequence	Protein name	Uniprot code-isoform	*m*/*z* heavy	*m*/*z* light
SM(ox)IR*PPY	AMELY	Q99218–1	889.4491	879.4393
SIR*PPYPSY	AMELX	Q99217–1	1089.5621	1079.5520

a R* = isotope labeled arginine , which induces a shift of +10 Da, and (Ox) = oxidation of methionine, which induces a shift of +15.99 Da.

MALDI-FTICR MS experiments were performed on a Bruker 9.4 T SolariX XR FTICR mass spectrometer controlled by FTMSControl software and equipped with a CombiSource and a ParaCell (Bruker Daltonics, Bremen, Germany). A Bruker Smartbeam-II Laser System was used for irradiation at a frequency of 1,000 Hz and using the “Minimum” predefined shot pattern. MALDI-FTICR spectra were generated from 500 laser shots in the *m*/*z* range from 600.01 to 5,000 with 1 M data points (i.e., transient length of 2.2020 s). Twenty spectra were averaged. The transfer time of the ICR cell was set to 1.2 ms and the quadrupole mass filter operating in RF-only mode was set at *m*/*z* 600.

MALDI-CASI-FTICR MS spectra were generated using parameters similar to those of MALDI-FTICR MS with some modifications. Fifteen spectra were averaged. Five ions were selected: *m*/*z* 829.4566 (SIRPPYP, AMELX protein, named AMELX1), 879.4393 (SM­(ox)­IRPPY, AMELY protein, named AMELYL), 889.4491 (SM­(ox)­IR*PPY, AMELY protein, named AMELYH), 1079.5520 (SIRPPYPSY, AMELX protein, named AMELX2L), 1089.5621 (SIR*PPYPSY, AMELX protein, named AMELX2H), with isolation windows at *m*/*z* 10. The acquisition time for each sample was less than 1 min.

### Peptide Identification

MS raw data from MALDI-FTICR were processed using DataAnalysis 5.0 software. The FTMS algorithm was employed with the following parameters: S/*N* > 4, Relative intensity threshold 0.01%, and Absolute intensity threshold 100. The list of peptide markers was searched using a homemade Python program (version 3.12.7, available on GitHub https://github.com/source-code-guru/SearchPPM). The list of [M + H]^+^ masses is indicated in the program, and the search was carried out with an error of 1 ppm. Log_2_ values for AMELX and AMELY intensities and heavy/light peptide ratios were calculated.

### LC-HRMS Double Validation

The remaining peptide solution of both modern adult (n = 18; 10 males, 8 females) and juvenile (n = 8; 4 males, 4 females) teeth, and archeological juvenile teeth from Rajhard (n = 43), previously analyzed by untargeted and targeted MALDI-FTICR MS, was subsequently used for LC-HRMS analysis. LC-HRMS analyses were performed on an Q Exactive Plus mass spectrometer hyphenated to an UltiMate 3000 RSLC Microfluidic HPLC System (Thermo Fisher Scientific, Waltham, Massachusetts, USA). The peptide solution (1 μL) was loaded onto an Acclaim PepMap 100 C18 precolumn (5 μm, 300 μm i.d. × 5 mm; Thermo Fisher Scientific) using solvent A (5% acetonitrile, 0.1% formic acid, *v/v*) for 3 min at a flow rate of 10 μL.min^–1^. Peptides were subsequently separated on an Acclaim PepMap 100 C18 analytical reversed-phase column (3 μm, 75 μm i.d. × 500 mm; Thermo Fisher Scientific) using a linear gradient of 5–40% solvent B (75% acetonitrile, 0.1% formic acid, *v/v*) at a flow rate of 250 nL.min^–1^. After peptide elution, the column was washed with 100% solvent B for 5 min before being re-equilibrated with solvent A. Both the pre- and analytical columns were maintained at 45 °C throughout the analysis. The total LC-HRMS run time was 140 min per sample.

LC-HRMS analyses were performed in positive ionization mode. Full MS scans were acquired in the Orbitrap mass analyzer over an *m*/*z* range of 350–1,500 at a resolution of 70,000 (at *m*/*z* 200), with the automatic gain control (AGC) target set to 1e6. Data-dependent MS/MS acquisition was performed using higher-energy collisional dissociation (HCD) on the 15 most intense precursor ions detected in each full MS scan. MS/MS spectra were acquired with an AGC target of 5e5 and a normalized collision energy (NCE) of 28. Dynamic exclusion was set to 90 s, and precursor ions with charge states of 1^+^ or greater than 8^+^ were excluded from fragmentation.

### LC-HRMS Data Validation

LC-HRMS raw files were processed with PEAKS (version v13, Bioinformatics Solutions Inc., Waterloo, Canada) against *Homo sapiens* enamel protein database containing 6 entries (COL1A1 (P02452), COL1A2 (P08123), AMELX (Q99217), AMELY (Q99218), Ameloblastin (Q9NP70), and Enamelin (Q9NRM1)). Unspecific digestion was selected and 10 ppm mass error for MS and MS/MS were applied. Methionine oxidation and proline hydroxylation (+15.99 Da), asparagine and glutamine deamidation (+0.98 Da), and phosphorylation of serine and threonine residues (+79.97 Da) were selected as variable modifications. A second database search was performed using MaxQuant (version 2.7.0.0) with the same search parameters. The resulting evidence.txt output file was subsequently processed using a Python script, Deamidation,^26^ to calculate peptide deamidation rates and estimate the conversion of asparagine to aspartic acid and glutamine to glutamic acid for each raw file.

## Results

### Development of Untargeted MALDI-FTICR MS Method on Modern Individuals

Biological sex estimation by mass spectrometry relies on the detection of sex-specific peptides derived from the amelogenin X (AMELX) and Y (AMELY) isoforms. Although LC-HRMS remains the most commonly applied approach for biological sex estimation in paleoproteomics,^2,3,7,11–13,16,27–29^ recent studies have also explored the use of Liquid Atmospheric Pressure Matrix-Assisted Laser Desorption/Ionization (LAP-MALDI-TOF) for this purpose.^9,10^ In the present study, MALDI-FTICR MS was selected for biological sex estimation based on two main advantages: (i) its high analytical throughput, as the MALDI source enables sample deposition on a target plate and acquisition of spectra within minutes; and (ii) the ultrahigh mass resolution of the FTICR analyzer, allowing the discrimination of peptides that differ by only a few millidaltons.

The untargeted MALDI-FTICR MS spectra obtained from modern male and female reference samples are presented in Supporting Information S1 (Figure S1). A large number of peptide signals were detected, corresponding to peptides derived from amelogenin X and Y (AMELX and AMELY), enamelin, and collagen proteins. A detailed view of the *m*/*z* range 800–1100 is shown in Supporting Information S1 (Figure S2). The AMELX-derived peptides SIRPPYP (*m*/*z* 829.4553), SIRPPYPSY (*m*/*z* 1079.5496), and SM­(oxidized)­IRPPY (*m*/*z* 879.4526) were detected. These peptides have previously been described as markers for amelogenin isoforms and are commonly used for biological sex estimation.^5,9,10,16,27^ Interestingly, among these potential markers, only the SM­(oxidized)­IRPPY peptide, specific to AMELY, was detected in the MALDI-FTICR MS spectra. This observation is consistent with previous LAP-MALDI-TOF analyses, where this peptide was also identified as the main AMELY marker. The nonoxidized SMIRPPY peptide from AMELY was detected at very low intensity, preventing reliable detection in the analyzed samples. Supporting Information S1 (Figure S3) provides an enlarged view of the corresponding mass region. Two peptide signals were observed between *m*/*z* 861 and 867: the M­(oxidized)­PLPPHPG peptide (*m*/*z* 861.4286), shared by AMELX and AMELY, and the SMIRPPY peptide (*m*/*z* 863.4441), specific to AMELY. The monoisotopic peak of SMIRPPY and the second isotopic peak of M­(oxidized)­PLPPHPG were found to be closely spaced, with a mass difference of only 0.009 *m*/*z*, highlighting the importance of the high mass resolution provided by the FTICR analyzer for accurate peptide assignment.

To validate the MALDI-FTICR MS results, LC-HRMS analyses were performed on modern reference samples from juvenile males (n = 4), juvenile females (n = 4), adult males (n = 10), and adult females (n = 8). The peptides identified by LC-HRMS in modern adult teeth, modern juvenile teeth, and archeological juvenile teeth (n = 43) from Rajhrad are listed in Supporting Information S3. The sequence coverage of AMELX and AMELY, together with the identified peptides, is presented in Supporting Information S1 (Figures S4 and S5) for representative modern samples (adult male (n = 1); adult female (n = 1); juvenile male (n = 1); juvenile female (n = 1)). As expected, no AMELY-derived peptides were identified in samples from adult and juvenile females. In contrast, several AMELY-specific peptides were detected in samples from adult and juvenile males. Among these, the peptide IRRPYSSY, phosphorylated at Y56, was identified in juvenile male by LC-HRMS. This phosphorylated peptide has previously been reported as a juvenile-specific AMELY marker^30^ but was not detected in the MALDI-FTICR spectra. Supporting Information S1 (Table S1) lists the peptides identified in modern juvenile, modern adult, and archeological samples. The identified peptides exhibited several post-translational modifications, including oxidation, deamidation, and phosphorylation. Comparison of the MALDI-FTICR spectra revealed clear differences between modern juvenile samples and the other groups (modern adults, archeological juveniles, and archeological adults) (Supporting Information S1, Figure S1). Several peptide signals detected in the latter groups were absent or markedly reduced in modern juvenile individuals. In particular, the AMELX- and AMELY-derived peptides commonly used for biological sex estimation (*m*/*z* 829, 879, and 1079) were detected at very low intensities in modern juvenile samples.

### Development of the High-Throughput Targeted MALDI-CASI-FTICR MS Method on Modern Individuals

Based on these data, the present method was designed to target three endogenous peptides derived from AMELX and AMELY that have previously been used for biological sex estimation (AMELX1, *m*/*z* 829.4553; AMELYL, *m*/*z* 879.4407; and AMELX2L, *m*/*z* 1079.5520).^10,27,28^ In addition, two isotope-labeled AQUA peptide standards corresponding to the peptides described by Koenig et al.^7^ (AMELYH and AMELX2H) were included as internal standards. Using the targeted MALDI-CASI-FTICR MS method, all five singly charged peptides, including the AQUA peptide standards, were selectively detected (Figure 1).

**Figure 1:**
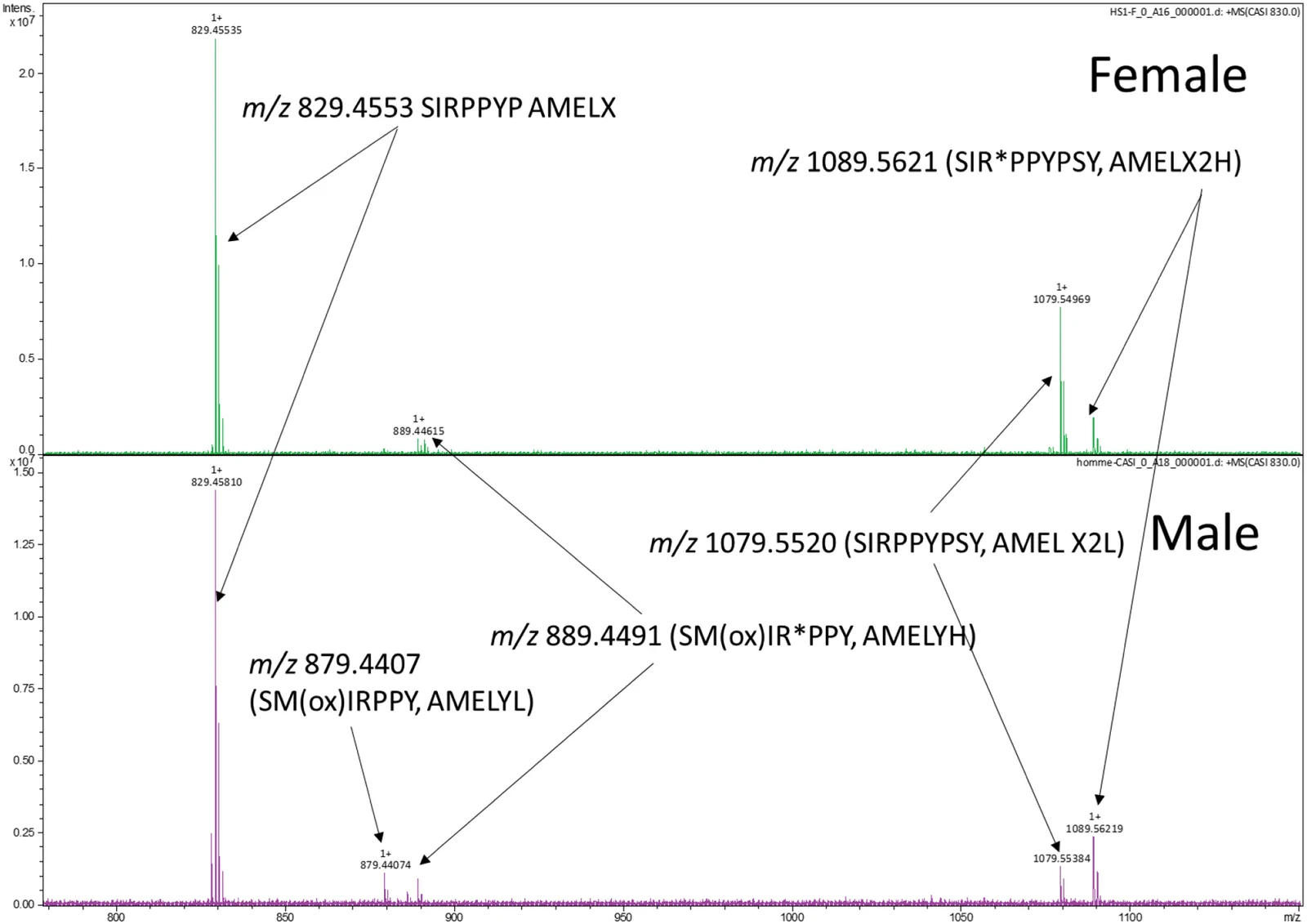
MALDI-CASI-FTICR mass spectra of modern male and female individuals. The peptides AMELX1, AMELX2, and AMELY, as well as the two AQUA peptides, are indicated. Heavy peptides are annotated with H and light peptides with L. The indicated peptides were monocharged.

As shown in Figure 1, the AMELX-derived peptides (AMELX1 *m*/*z* 829.4553 and AMELX2L 1079.5520) were detected in both female and male samples, whereas the AMELY-derived peptide (AMELYL *m*/*z* 879.4407) was detected exclusively in the male sample. The isotope-labeled internal standards were detected at *m*/*z* 889.4491 (AMELYH) and *m*/*z* 1089.5621 (AMELX2H).

The MS spectra of modern male references obtained with MALDI-CASI-FTICR and MALDI-FTICR MS are shown in the Supporting Information S1, Figure S5A. Zooming in on the region containing the peptides of interest shows that the CASI method allows only the selected peptides to be detected and facilitates their identification (Supporting Information S1, Figure S5 B). With this targeted method, data analysis allows for simpler and faster identification of amelogenin peptides of interest. Numerous studies have shown that these peptides can be used to study the teeth of adults or juveniles.^3,13^

A dilution series of AQUA Peptides in a pool of five modern male extraction solutions ranging from 0.3 to 600 fmol was analyzed using the MALDI-CASI-FTICR MS method and showed good linearity (R^2^ = 0.989 for AMELY and R^2^ = 0.997 for AMELX) (Figure 2 A, B). To perform quantitative analysis, it is important to determine the limit of detection (LOD) or sensitivity of the method, and the limit of quantification (LOQ) of each targeted peptide. The limit of detection signifies the lowest signal that can be detected, whereas the limit of quantification denotes the lowest MS signal that can be reliably quantified. The LOD for each peptide was 0.6 fmol, and the LOQ was 3 fmol. For the analysis of archeological samples, each AQUA Peptides was spiked at 6 fmol to enable effective detection without affecting the detection of native peptides. Regarding the data on LOD and LOQ, our results are similar to those obtained by LC-HRMS and published by Koenig et al.^7^

**Figure 2:**
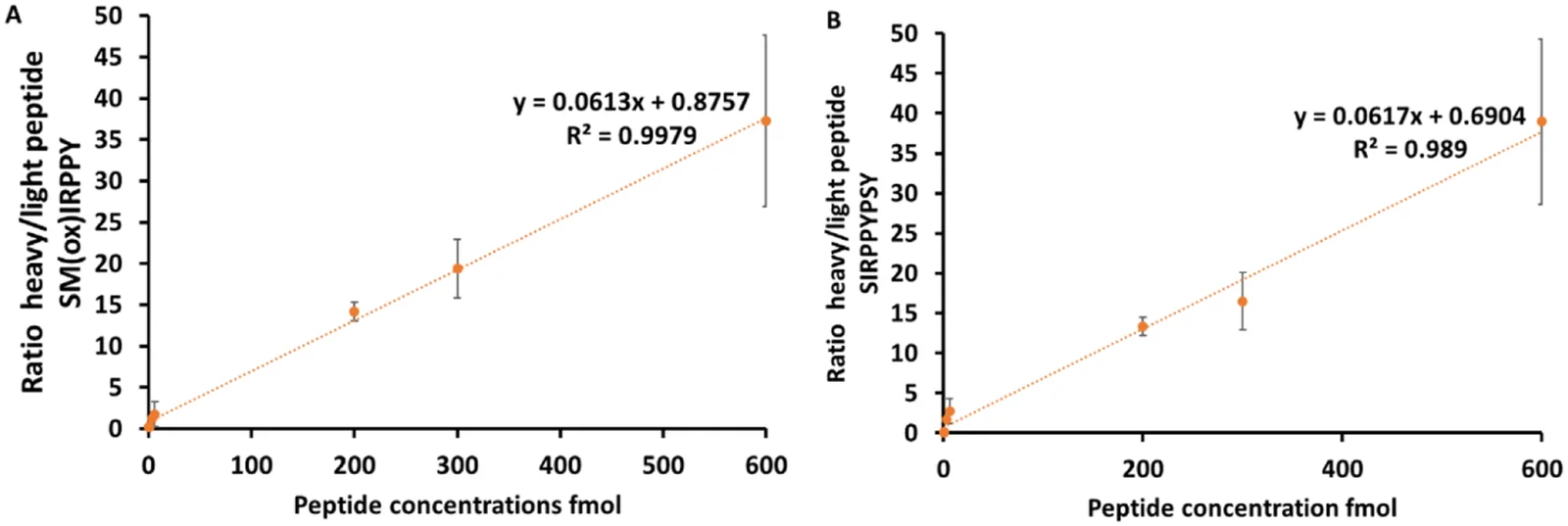
Dilution series of SM­(ox)­IRPPY, AMELYH (A), and SIRPPYPSY, AMELX2H (B) peptides ranging from 0.3 to 600 fmol in a pool of five modern male extraction solutions using a MALDI-CASI-FTICR MS targeted method. All analyses were performed in triplicate. For each linear regression, the equation and R^2^ are indicated. Black bars correspond to the standard deviations.

Using our modern male reference samples, we observed a linear correlation between Log_2_ AMELX2L (*m*/*z* 1079.5520) and Log_2_ AMELY (*m*/*z* 879.4393), with a Pearson correlation of 0.641 (p-value = 0.001). We observed a better linear correlation between Log_2_ AMELX1 (*m*/*z* 829.4566) and Log_2_ AMELY (*m*/*z* 879.4393), with a Pearson correlation of 0.731 (p-value = 0.0001) (Figure 3).

**Figure 3:**
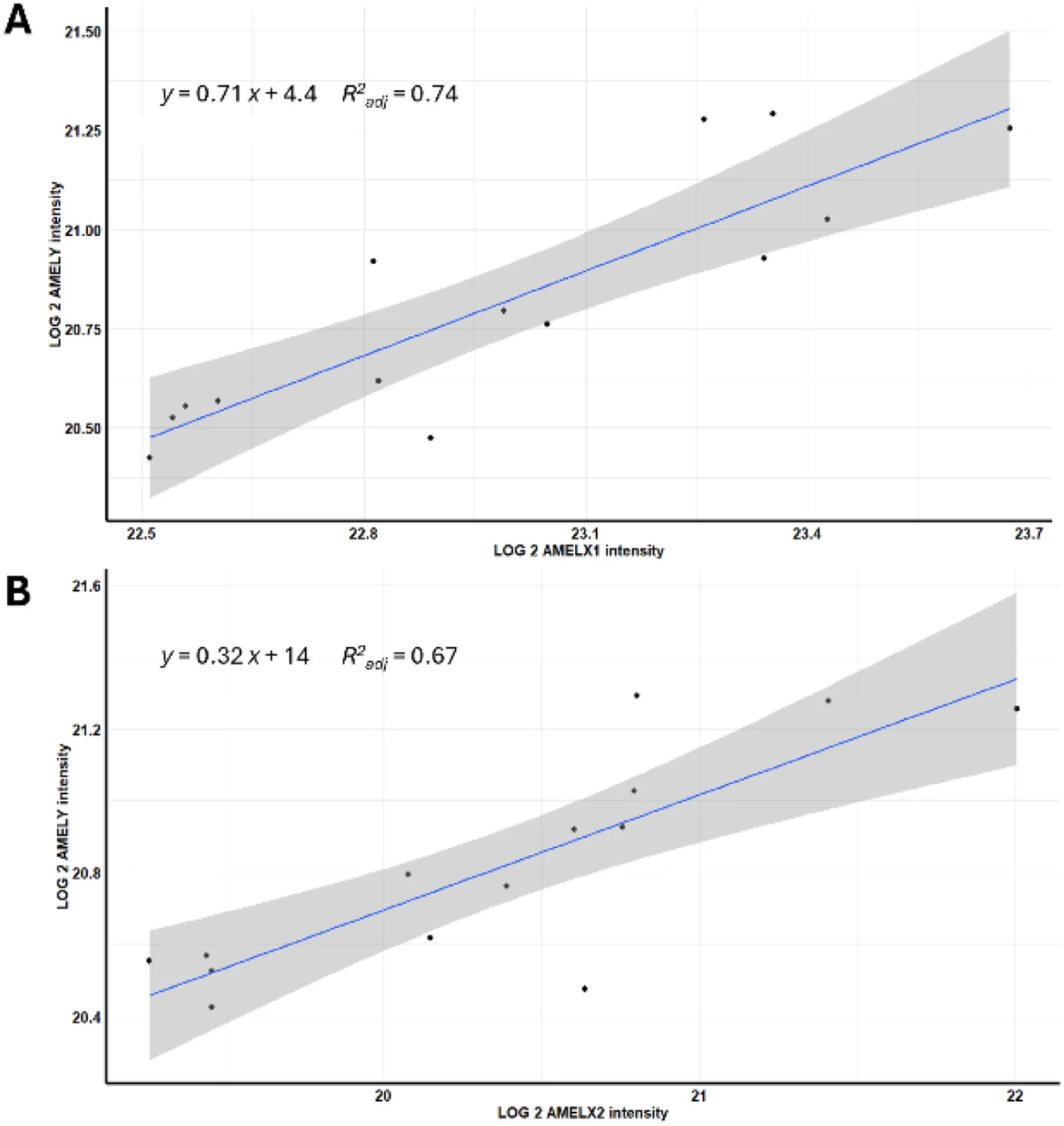
Linear regression of Log_2_ AMELY intensity as a function of Log_2_ AMELX1 intensity (A) or AMELX2L (B). This study involved the analysis of 14 modern adults males using MALDI-CASI-FTICR. Blue line = linear regression model; gray line = confidence interval (observed values 95% (±2σ)); black dot = sample.

As shown in Figure 3A, four adult male samples fall outside this interval, whereas three adult male samples do so in Figure 3B. Using MALDI-CASI-FTICR MS, biological sex was estimated in modern adult individuals based on the Log_2_ AMELY intensity together with either the Log_2_ AMELX1 or Log_2_ AMELX2 intensity (Supporting Information S1, Figure S7). In the modern adult validation cohort, all 14 male and 14 female individuals were correctly classified. This corresponds to a sensitivity of 100% (14/14; 95% CI: 76.8–100%), a specificity of 100% (14/14; 95% CI: 76.8–100%), and an overall accuracy of 100% (28/28; 95% CI: 87.7–100%), calculated using the exact Clopper-Pearson binomial method. These results are consistent with those reported by Koenig et al.,^7^ who also achieved complete correct sex assignment in a validation cohort of modern adult individuals. However, despite the absence of misclassifications in our adult cohort, the confidence intervals remain relatively broad because of the limited sample size. Consequently, the present data set should be considered a proof-of-concept validation, and larger cohorts will be required to obtain more precise estimates of diagnostic performance. In contrast, performance was lower in juvenile individuals: biological sex could be estimated for only 50% of the samples, with correct identification achieved for two of four juvenile males, whereas Koenig et al.^7^ correctly identified 9 of 10 modern juvenile males (90%). As the performance observed in juvenile individuals differs between the two studies, larger cohorts of modern juvenile male teeth will therefore be required to robustly assess the specificity of the proteomics-based approach.

The Log_2_ ratio of intensities for the peptide *m*/*z* 889.4491 (SM­(ox)­IR*PPY, AMELY protein, named AMELYH)/*m*/*z* 879.4393 (SM­(ox)­IRPPY, AMELY protein, named AMELYL), and *m*/*z* 1079.5520 (SIRPPYPSY, AMELX protein, named AMELX2L)/*m*/*z* 1089.5621 (SIR*PPYPSY, AMELX protein, named AMELX2H) were calculated and plotted on a graph (Supporting Information S1, Figure S8). The results showed a better correlation with the intensities of AMELY and AMELX1. The threshold values were determined: Log_2_ AMELX < 18 and Log_2_ AMELY < 17. Considering the ratio of light to heavy peptides, if the ratio 889/879 > 10, the sample was considered indeterminate. The 1089/1079 ratio showed a wide variation in intensity. Samples with a 1089/1079 ratio >120 were considered indeterminate. Using our method, heavy peptides were employed as internal standards, allowing the detection threshold to be established and thereby minimizing the risk of false-negative results (i.e., incorrectly classifying a male sample as female).

To estimate biological sex while accounting for both Log_2_ AMELY and AMELX1 values, the correlation line derived from modern male adults and juveniles was used. Figure 4 shows the resulting correlation line based on the modern male reference samples.

**Figure 4:**
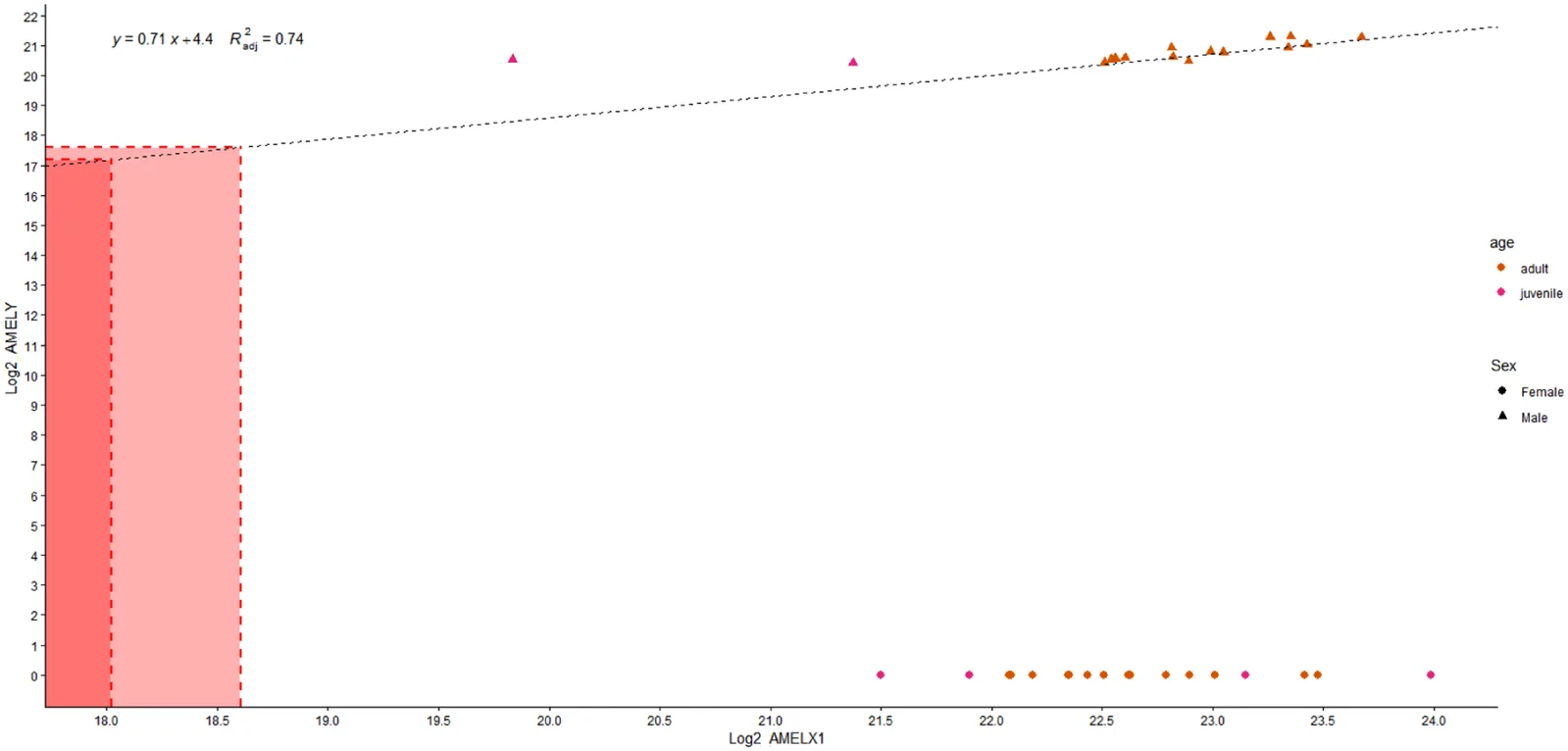
Biological sex estimation of modern teeth by MALDI-CASI-FTICR, showing Log_2_ AMELY intensity as a function of Log_2_ AMELX1 intensity. The dotted line represents the correlation line derived from modern adult male samples (y = 0.71x + 44; adjusted R^2^ = 0.74). The dark red shaded area indicates the limit for sex estimation at a noise threshold of 150,000, whereas the light red shaded area corresponds to a noise threshold of 200,000.

Two of the four modern juvenile male samples were identified as outliers and were therefore excluded from the correlation analysis. Based on the background noise intensity measured in the spectra, an exclusion zone for biological sex estimation was defined. Two background noise thresholds were evaluated: 150,000 and 200,000. For the 150,000 threshold, the exclusion limits were log_2_ AMELY = 17.194 and log_2_ AMELX = 17.179. For the 200,000 threshold, the exclusion limits were log_2_ AMELY = 17.609 and log_2_ AMELX = 17.827.

### Biological Sex Estimation of Archeological Adult and Juvenile Teeth

Both high-throughput untargeted and targeted proteomics MALDI-FTICR MS and MALDI-CASI-FTICR methods were performed on 130 remaining samples from medieval Mikulčice and Rajhrad in Great Moravia. Based on the heavy/light peptide ratio, both methods led to the identification of 60 females, 69 males, and 1 indeterminate individual (Supporting Information S1, Figure S9). The heavy/light peptide ratios for all samples are shown in Supporting Information S1, Figure S10, and Supporting Information S2, Tables S1, S2.

To improve the accuracy of biological sex estimation in archeological individuals and identify indeterminate cases, a scatter plot of Log_2_ AMELY intensity versus Log_2_ AMELX1 intensity was generated (Figure 5). The plot includes all modern reference samples (adults and juveniles), with the correlation line fitted to the male individuals.

**Figure 5:**
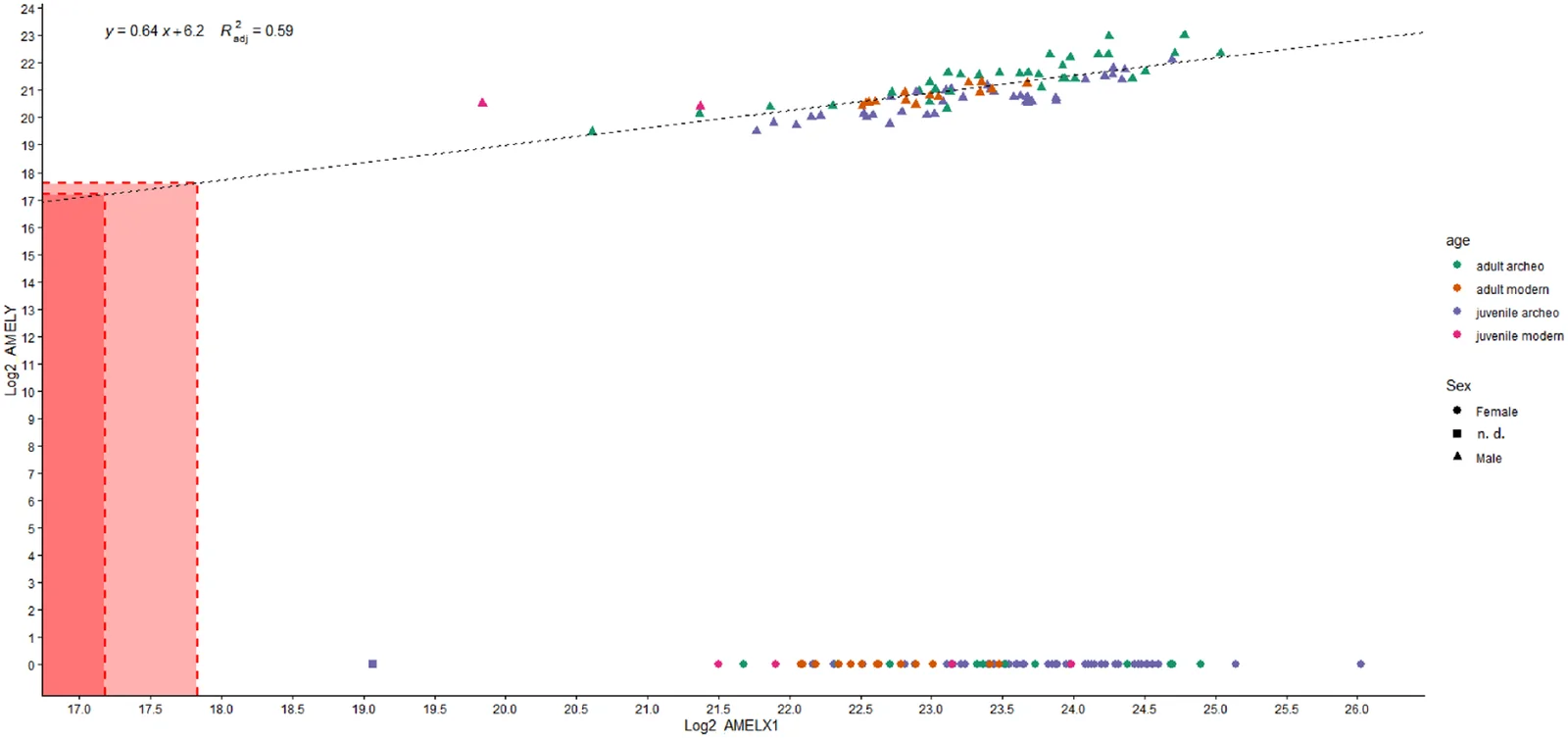
Log_2_ AMELY intensity plotted as a function of Log_2_ AMELX1 intensity for samples from the Mikulčice and Rajhrad archeological sites, together with the modern reference samples. The black dotted line represents the correlation line for modern male individuals (y = 0.64x + 6.2; adjusted R^2^ = 0.59). The red dotted lines indicate the biological sex estimation thresholds calculated using FTICR background noise levels of 150,000 (first red dotted line) and 200,000 (second red dotted line). Exact values are reported in Supporting Information S4.

A correlation line was fitted using modern male individuals, yielding the equation *y* = 0.64*x* + 6.2 with an adjusted R^2^ of 0.59. To define the validity range of the biological sex estimation method, two FTICR background noise thresholds (150,000 and 200,000) were considered. These thresholds corresponded to Log_2_ AMELY and Log_2_ AMELX intensities of 17.1 and 17.1 for a background noise level of 150,000, and 17.6 and 17.8, respectively, for a background noise level of 200,000. Based on these values, an indeterminate range was established, within which biological sex estimation was considered nonconclusive.

The two included modern juvenile males fell within the same distribution as the archeological samples (Figure 5, pink triangles), indicating that the correlation established from modern male individuals is also applicable to juvenile samples. Sample H163 clustered with the female individuals (Log_2_ AMELX1 = 19.06). However, its values differed markedly from those of the other samples. Therefore, LC-HRMS analysis is required to confirm its sex estimation.

### Comparison of Proteomics Biological Sex Estimation with Sex Assessments Obtained by Conventional Methods

The sex of 30 adult individuals from Mikulčice was originally assessed by Stloukal^19,20^ based on their skeletal morphological traits. Proteomics sex estimation was consistent with these assessments, except for three individuals (H392, H493, and H1082), for whom the proteomics results indicated the opposite sex (Table 2).

**Table 2:** Morphological Sex Assessment of 30 Adult Individuals from Mikulčice Was Performed by Stloukal (1963, 1967), Brůžek and Velemínský (2017), and Zazvonilová et al. (2020), and Proteomics Sex Estimation Was Performed in the Present Study^a^

		Sex assessment/Sex estimation						
		Osteomorphology			Proteomics			
Grave number	Age (years) Stloukal^19,20^	Stloukal^19,20^	Brůžek, Velemínský 2017 -unpublished	Zazvonilová et al.^21^	Tooth class	Log_2_ AMELX	Log_2_ AMELY	This study
H362	40–50	M	M	?	I_2_	21,13	19,01	M
H369	40–50	F	F	?	_2_I	20,94	0,00	F
H392	40–50	M?	-	?	M	23,70	0,00	F
H401	50–60	M	?	?	P_2_	22,82	20,81	M
H423	40–50	M	M	?	P^2^	23,33	21,77	M
H425	40–59?	M		?	_1_P	23,94	22,19	M
H426	40–50	M	M	?	C	23,00	21,09	M
H429	20–39	M?	-	?	M	23,04	21,54	M
H439	40–50	M	-	?	^2^P	23,59	21,60	M
H483	40–50	M	-	?	P^2^	23,80	22,08	M
H484	50–60	F	-	?	_2_P	23,55	0,00	F
H487	40–50	M?	-	?	C	23,30	21,47	M
H493	40–50	F?	-	?	M	23,07	21,28	M
H495	40–50	M?	M?	?	C	23,61	21,67	M
H505	20–30	F	F	?	_1_P	22,41	0,00	F
H507	50–60	M	-	?	^3^ M	23,27	22,21	M
H512	adult	F?	-	?	_2_P	22,81	0,00	F
H551	40–59	M	-	?	C	23,07	21,17	M
H553	20–30	M	-	?	C	23,25	21,57	M
H563	40–50	M	M	?	C	21,82	20,23	M
H565	20–30	M?	-	?	I^2^	23,94	21,59	M
H588	adult	F?	-	?	M	24,00	0,00	F
H589	40–59?	M	-	?	M_3_	23,10	21,69	M
H605a	14–19	M	-	-	^2^I	23,37	21,16	M
H626	20–30	F	-	-	_2_M	23,17	0,00	F
H628	40–50	M?	-	M	M	24,15	21,98	M
H697	50–60	F	-	?	^2^ M	23,23	0,00	F
H701	20–30	M	M	?	C	21,80	20,24	M
H1082	adult	F	F	?	C	22,51	20,20	M
H1182	adult	F?	F?	F	C	23,60	0,00	F

a Tooth nomenclature: I = incisor, C = canine, *P* = premolar, M = molar, upper index = upper jaw, lower index = lower jaw, side of the index indicates the side of the tooth (left or right). A capital letter indicates permanent teeth, and a lowercase letter indicates deciduous teeth. A question mark indicates individuals for whom Zazvonilová et al. (2020) stated that sex could not be assessed based on the methods used in the study.

In the case of nonadult individuals from Rajhrad—whose sex is generally not assessed using traditional osteological methods due to the absence of fully developed sexually dimorphic skeletal features—Hanáková et al.^23^ did not perform sex assessment. However, Pospíšilová^24^ assessed the sex of nonadult individuals from Rajhrad using a metric analysis of permanent teeth. Among the 13 individuals shared between her study and the present work, we proteomically confirmed the sex of 11 individuals (Table 3).

**Table 3:** Metric Sex Assessments of 13 Juvenile Individuals from Rajhrad Based on Permanent Teeth by Pospíšilová (2017) and Proteomic Sex Estimation of 100 Juvenile Individuals Performed in the Present Study^a^

Grave number	Age (years) Hanáková et al.^23^	Tooth metric sex assessments Pospíšilová^24^	Tooth class	Log_2_ AMELX	Log_2_ AMELY	Proteomics This study
H2	6		^1^ M	21,72	19,75	M
H5	11		_1_m	22,22	18,83	M
H11	16–17		M	23,72	0,00	F
H20	7–8		M_1_	22,56	20,55	M
H21	10		^2^ M	23,35	21,56	M
H23	3		M_1_	22,46	20,26	M
H24	3		^1^m	22,41	19,50	M
H25b	10		M	21,52	19,21	M
H26	6		^1^ M	22,33	0,00	F
H27	4		m_2_	23,08	0,00	F
H28	4		m^2^	22,29	19,88	M
H29	3–4		m_1_	23,08	20,58	M
H32	2.5–3		^2^m	21,86	19,44	M
H36	6		_1_M	21,88	20,10	M
H41	4		M^1^	21,82	0,00	F
H43	16–18		M	23,95	21,99	M
H50	10		_1_M	23,39	0,00	F
H52	16?	M	M_2_	21,81	20,01	M
H54	15–16		_2_M	22,90	0,00	F
H55	4		_1_M	22,64	0,00	F
H59	11		M_2_	21,40	18,85	M
H64	12		_2_M	21,14	0,00	F
H65	7		_1_M	23,22	0,00	F
H65a	2.5		M_1_	22,80	0,00	F
H66	14–15		M	22,19	19,42	M
H76	8	M	M	20,95	18,43	M
H77	15–20	M	M_2_	21,02	19,17	M
H78	11	F	M^1^	21,41	0,00	F
H79	9		_1_M	21,25	18,87	M
H80	6–7		_1_M	21,37	17,83	M
H80a	3		m^1^	22,96	0,00	F
H81	12	F	M_1_	22,34	0,00	F
H85	8		_1_M	22,40	20,00	M
H86	11		^1^ M	19,91	17,77	M
H86a	10–11		_1_M	22,18	20,48	M
H87	4		M1	21,46	19,40	M
H88	2		^2^m	22,00	0,00	F
H91	7–8		_1_M	21,99	0,00	F
H95	14	M	_1_M	23,44	0,00	F
H96	2–3		^2^m	22,31	0,00	F
H98	2.5		m^1^	20,83	0,00	F
H99	8		_1_M	23,56	0,00	F
H103	7–8	M	_1_M	21,85	19,96	M
H106	15–16	F	M^2^	22,62	0,00	F
H108	10		M2	21,59	0,00	F
H111	10	F	M^1^	22,80	0,00	F
H113	6		^2^m	23,13	0,00	F
H118	14–15		M^1^	23,57	0,00	F
H119	11		M_2_	21,52	19,97	M
H120	15–16		M_2_	25,54	0,00	F
H121	6		M_1_	21,94	0,00	F
H122	16–17		M_2_	23,43	21,03	M
H124	9		M_1_	22,49	0,00	F
H126	2.5		m_2_	23,05	20,76	M
H127	17–18		M_3_	21,83	19,73	M
H129	2		M1	22,04	0,00	F
H133	18–20		M_2_	23,40	21,42	M
H134	8		M^1^	22,86	0,00	F
H134a	2.5		*c	21,33	0,00	F
H141b	15–19		^1^ M	22,68	0,00	F
H145	6		_1_M	23,21	0,00	F
H146	7		M	21,85	19,35	M
H151	18–20		M_3_	22,81	20,92	M
H155	13		M_2_	23,67	21,47	M
H159	18–20		_1_M	22,60	20,57	M
H160	5		_1_M	22,99	20,79	M
H162	5		^1^ M	22,82	20,62	M
H163	3		m_1_	19,31	0,00	n.d.
H165	11		_2_M	22,72	0,00	F
H166	5		M_1_	20,68	0,00	F
H167	not provided		C	22,81	0,00	F
H168	9		M	21,96	0,00	F
H169	2–2.5		m_1_	20,77	0,00	F
H171	7		M_1_	23,23	0,00	F
H175	13	M	M	23,54	0,00	F
H176	10		^2^ M	22,45	0,00	F
H177	11–12	M	_2_M	20,65	18,38	M
H182	11		_1_M	23,70	0,00	F
H183	11		_2_M	23,19	0,00	F
H184	4–5		m_2_	22,51	0,00	F
H185	3		m	22,78	0,00	F
H186	not provided		^1^ M	23,41	0,00	F
H187	8	F	M_1_	22,21	0,00	F
H188	6		M_1_	22,56	20,35	M
H189	2.5		m^2^	21,58	18,88	M
H191	4–5		M1	22,79	19,95	M
H192	12–13		P	22,96	20,37	M
H193	14		_1_M	23,78	0,00	F
H194	16		M_2_	23,34	20,93	M
H196	12	F	_2_M	24,22	0,00	F
H201	18–20		M	23,26	21,28	M
H202	4–5		M1	21,30	19,33	M
H209	10		_2_M	22,10	19,78	M
H210	7		M	22,51	20,43	M
H220	17–18		^3^ M	22,02	19,68	M
H225	16–18?		_1_M	23,12	0,00	F
H234	17–18		M	23,95	0,00	F
H238	16–18		M	22,89	20,48	M
H241b	13		M	23,65	0,00	F
H243	6		_1_M	22,54	20,53	M

a Tooth nomenclature: C = canine, M = molar, upper index = upper jaw, lower index = lower jaw, and side of the index indicates the side of the tooth (left or right). A capital letter indicates permanent teeth, a lowercase letter indicates deciduous teeth. n.d. = not determined.

To further investigate the undetermined sample H163, an LC-HRMS analysis was performed following the MALDI-FTICR analysis. The proteins AMELX, AMELY, COL1A1, COL1A2, ameloblastin, and enamelin were identified. A total of 89 peptides were assigned to AMELX and 49 to AMELY. The sequence coverage obtained for AMELX (residues 41–52) and AMELY (residues 55–77) is presented in Figure S11 (Supporting Information S1). Within the sex-specific region, only peptides corresponding to the AMELX sequence were detected (18 peptides), whereas no AMELY-specific peptides were identified. These findings support a female sex estimation for sample H163.

### Blanks, Deamidation, and Peptide Length

Analysis of blank samples by both MALDI-FTICR and LC-HRMS did not reveal the presence of peptides derived from enamel proteins, supporting the absence of detectable contamination during sample preparation and analysis.

The extent of deamidation was assessed using the Python script developed by Mackie et al.^26^ Deamidation levels were calculated for AMELX, AMELY, COL1A1, and COL1A2 (Figures S12–S15, Supporting Information S1). For all four proteins, deamidation levels were low in modern juvenile samples and higher in modern adult samples. A comparison between modern and archeological juveniles showed an increase in deamidation levels in archeological individuals. However, the difference between modern and archeological adults was less pronounced. Among the analyzed proteins, AMELY and COL1A2 displayed slightly higher deamidation levels compared with the other proteins.

Regarding peptide length, consistent with previous observations in other archeological contexts,^31,32^ archeological samples exhibited the shortest identified peptides, with an average molecular mass of 1169 Da, compared with 1309 Da for modern juveniles and 1205 Da for modern adults (Figure S16, Supporting Information S1).

## Discussion

The MALDI-FTICR mass spectrometer used in this study has the highest resolution compared to other mass spectrometers currently used for biological sex estimation and is becoming the technique of choice. Its MALDI source has the advantage of not using chromatography, which drastically reduces the analysis time and estimated costs. The combination of high-throughput and targeted approaches can confidently estimate the biological sex of up to 1440 individuals per day and represents the most efficient method to date. The use of synthetic isotope-labeled peptide (AQUA Peptides) standards allowing for greater consistency and reproducibility between analyses and laboratories (Table 4).

**Table 4:** Comparison of Currently Available Methods for Biological Sex Estimation by Proteomics^a^

Analytical technique	Sampling, acid nature and concentration (conc. eq in %), time	Analysis (acquisition)	Standards (internal/external)	Analysis time (eq sample-per-day)	Resolution*	Estimated costs	Citation
LC-QTOF	Acid etching, 1.4 M HCl (5%), 2 min	Untargeted (DIA)	None	120 min (12 SPD)	Not described, 40,000 – 60,000 estimated	+++	^3^
LC-Orbitrap	Acid etching, 1.4 M HCl (5%), 2 min	Untargeted (DDA)	None	65 min (22 SPD)	120,000 at *m*/*z* 200	+++	^13^
LC-Orbitrap	Enamel powder (10–50 mg)	Targeted (PRM)	Synthetic isotope-labeled peptides (internal)	110 min (13 SPD)	120,000 at *m*/*z* 200	+++	^16^
LC-Orbitrap	Acid etching 1 M TFA (10%), 2–10 min	Targeted (PRM)	Synthetic peptides (external)	7.2 min (200 SPD)	60,000 at *m*/*z* 200	++	^7^
LC-Orbitrap	Acid etching, 1.4 M HCl (5%), 2 min	Targeted (PRM)	None	3 min (480 SPD)	70,000 at *m*/*z* 200	+	^12^
LC-Orbitrap	Enamel powder (100 mg)	Targeted (MRM)	None	120 min (12 SPD)	Not described, 60,000 at *m*/*z* 400 estimated	+++	^15^
MALDI-TOF	Acid etching, 2.9 M HCl (10%), 5 min	Untargeted (DDA)	None	Not described, 1 min (1440 SPD) estimated	Not described, 20,000 estimated	+	^17^
LAP-MALDI-TOF	Acid etching, 2.9 M HCl (10%), 5 min	Untargeted (not described)	None	1 min (1440 SPD)	Not described, 40,000 – 60,000 at *m*/*z* 956 estimated	+	^9^
MALDI-FTICR	Acid etching, 0.6 M HCl (2.2%), 1h	Targeted (CASI)	Synthetic isotope-labeled peptides (internal)	1 min (1440 SPD)	1,000,000 at *m*/*z* 200	+	This study

a Costs were estimated based on chemical consumption (nature and concentration) for sample preparation and analysis, consumables used during sample preparation (Eppendorfs, sample plate, etc.) and analysis (analytical column, pump, etc.), analysis time consumption (operating), and environmental impact (electricity and gas consumption). The cost estimation is based on Europe. Resolution*: if the resolution is not described in the publication, the value estimated by the mass spectrometer manufacturer is annotated.

Costs were estimated based on chemical consumption (nature and concentration) for sample preparation and analysis, consumables used during sample preparation (Eppendorfs, sample plate, etc.) and analysis (analytical column, pump, etc.), analysis time consumption (operating), and environmental impact (electricity and gas consumption). The cost estimation is based on Europe. Resolution*: if the resolution is not described in the publication, the value estimated by the mass spectrometer manufacturer is annotated.

The use of the CASI acquisition method with a Fourier transform ion cyclotron resonance (FTICR) mass spectrometer provides a low detection limit.^33^ It has been shown that the CASI method yields results similar to those obtained using LC–HRMS for the analysis of archeological bone samples.^34^ We used two peptides, *m*/*z* 889.4491 (SM­(ox)­IR*PPY) for the AMELY isoform and *m*/*z* 1089.5621 (SIR*PPYPSY) for the AMELX isoform, as standards from the publication by Koenig et al.^7^ The heavy/light peptide ratio allows us to eliminate samples with too low an intensity for light or heavy peptides. The use of these peptides improves the confidence in biological sex estimation.^7^ It has been shown that MALDI allows for extremely accurate ratio measurements, which are similar to those obtained by MRM analysis.^35^ Adding a heavy peptide for the mass *m*/*z* 829.4590 would be useful for improving confidence in the results. The unique disadvantage of this method is the high cost of synthetic isotope-labeled peptides used for internal quantification (approximately €500 per peptide) compared to the synthetic peptides used for external quantification (approximately €150 per peptide).

The differences observed between MALDI and ESI in the detection of peptides carrying post-translational modifications (PTMs) may be explained by several factors.^36^ MALDI and ESI rely on fundamentally different ionization mechanisms, which can influence the preservation and detection of labile modifications. During MALDI analysis, ionization is initiated by irradiation of a solid matrix-analyte mixture with an ultraviolet laser, resulting in the transfer of substantial internal energy to the molecules. Common matrices such as CHCA (α-cyano-4-hydroxycinnamic acid) are considered relatively “hot” matrices and may promote the fragmentation of labile modifications during the ionization process. In particular, phosphorylated peptides can undergo neutral loss of phosphoric acid (−97.98 Da) or phosphate (−79.97 Da) prior to detection, leading to reduced signals for intact phosphorylated species.^37^ In contrast, ESI is considered a softer ionization technique performed in the liquid phase under atmospheric pressure conditions, limiting the energy transferred to analyte molecules and favoring the preservation of labile PTMs. Furthermore, MALDI predominantly generates singly charged ions ([M + H]^+^) in positive mode. Because phosphorylation increases peptide acidity, phosphorylated peptides may exhibit reduced ionization efficiency due to their lower ability to acquire a proton compared with their unmodified counterparts. Consequently, their detection sensitivity can be substantially decreased.^38^ Another factor contributing to the lower detection of modified peptides by MALDI is the absence of chromatographic separation prior to ionization. In this context, highly abundant unmodified peptides may compete with low-stoichiometry modified peptides during ionization, resulting in signal suppression.^39^ Conversely, when LC is coupled to an ESI source, modified peptides can be separated from their unmodified counterparts based on altered physicochemical properties, including changes in hydrophobicity caused by phosphorylation or oxidation. This separation modifies peptide retention times and reduces ionization competition, thereby improving the detection of modified peptide species.^40^

It is important to note that the biological methods for estimating the sex of individuals has weaknesses. (i) It should be emphasized that female sex estimation carries the highest risk of false positives when data quality is low and AMELY signal intensity is weak. In 1992, Salido et al.^41^ have reported that transcription of amelogenin gene from the human Y chromosome may account for only approximately 10% of total amelogenin transcription. In 2019, Parker et al.^28^ demonstrated that a quantitative proteomics approach improves the estimation of false-positive rates and proposed a probabilistic method for sex estimation. However, the authors also highlighted the limitations of this approach, particularly due to the weak AMELY signal. More recently, in 2024, Koenig et al.^7^ developed a quantitative method based on LC-HRMS, incorporating a statistical framework specifically designed for the positive identification of female individuals. In this context, validation thresholds were applied in the present study to reduce false positives, relying on internal standards to strengthen the robustness of interpretations. (ii) Sex estimation by mass spectrometry also faces an additional limitation related to the possible deletion of the Y-chromosomal amelogenin gene. Such deletions have been reported in populations worldwide. Although this event remains rare—generally occurring in fewer than 1% of individuals and often well below 0.1% ^42,43^ —significantly higher frequencies have been documented in biological males from certain populations of the Indian subcontinent. In these cases, the absence of AMELY may lead to the misclassification of XY individuals as female when relying solely on proteomics detection. In addition, other biological conditions affecting the sex chromosome complement—such as sex chromosome trisomies, including Klinefelter syndrome (XXY)—may influence the results. These biological variations constitute important constraints that must be taken into account and highlight the need to interpret proteomics sex estimation within a broader genetic and anthropological context.

### Proteomics Sex Estimation on Juvenile Individuals

From a biochemical perspective, the amelogenin proteins preserved in deciduous and permanent enamel contain the same sexually dimorphic peptide sequences derived from AMELX and AMELY. Consequently, the molecular basis for proteomics sex estimation is identical in juveniles and adults. Nevertheless, several structural and developmental differences between deciduous and permanent enamel may influence protein preservation and peptide recovery. Deciduous enamel is generally thinner and slightly less mineralized than permanent enamel.^44^ Despite these differences, the highly mineralized enamel matrix effectively entraps residual enamel proteins and provides long-term protection against degradation, allowing the recovery of sex-informative peptides even in archeological individuals. Amelogenin is a transient extracellular matrix protein that is secreted in large amounts during amelogenesis and progressively cleaved by proteases as enamel matures.^45^ As a result, mature enamel in both deciduous and permanent teeth retain only trace amounts of amelogenin-derived peptides. Nevertheless, advances in mass spectrometry sensitivity enable the detection of these residual peptides in both deciduous and permanent enamel.

Ziganshin et al.^30^ further demonstrated that juvenile enamel may preserve developmental information in addition to sex-specific peptides. In their study, two AMELY-specific peptides were identified in male individuals, including the peptide SM­(ox)­IRPPYS, which was detected in both children and adults. In contrast, the phosphorylated peptide IRPPYSS­(phospho), corresponding to phosphorylation of S66, was detected exclusively in deciduous teeth. This observation is consistent with the role of S66 phosphorylation during amelogenesis and suggests that some developmental PTMs may be preferentially preserved in juvenile enamel. The feasibility of proteomics sex estimation in juveniles has also been demonstrated by several recent studies. Parker et al.^28^ first established the applicability of paleoproteomics sex estimation using archeological deciduous teeth dating to approximately 7,300 years before present. More recently, Adair et al.^10^ showed that LAP-MALDI-MS enables rapid sex estimation from medieval deciduous teeth in less than 10 min per sample without prior liquid chromatographic separation. In addition, Morvan^46^ developed the *SexPeptID* software to reanalyze paleoproteomics data sets spanning from the Pleistocene to modern populations including juveniles. Her work emphasized the importance of stringent statistical criteria for sex estimation to minimize false-positive male identifications resulting from contamination or low-intensity AMELY signals. Together, these studies demonstrate that proteomics sex estimation is applicable to juvenile individuals and highlight the value of combining highly sensitive mass spectrometry with rigorous data interpretation to improve the reliability of sex estimation in archeological remains.

### Comparison of Morphological versus Proteomics Sex Estimation

With regard to the comparison of morphological sex assessment performed by different authors and biological sex estimation by proteomics analysis, the following observations can be made.

For adult individuals from the Mikulčice site, sex was assessed for all individuals in our sample (n = 30) by Stloukal in the 1960s^19,20^ using the methods available at that time.^47–50^ Remarkably, despite the lack of sophisticated methods available today, only three out of the 30 cases (H392, H493, and H1082) were assigned a sex different from that estimated by proteomics analysis in the present study.

Regarding subsequent revisions, Zazvonilová et al.^21^ however, they were able to assess the sex of only two individuals from our sample. For the remaining individuals, the application of their selected methods^51–55^ was not possible. Their approach relied primarily on the pelvic bone, with the skull used preferentially in cases where the pelvis was not preserved.

Brůžek and Velemínský assessed sex using primary and secondary sex diagnosis^22^ for only a subset of individuals from Mikulčice, with 11 individuals overlapping with our sample. In one case, sex was not assessed, and the individual was marked with a question mark, whereas in another case (H1082), sex was assigned differently from that subsequently confirmed by proteomics analysis.

Notably, in one individual from our sample (H1082), both Stloukal^19,20^ and Brůžek with Velemínský—each highly experienced observers—confidently assigned the same sex, which was shown by proteomics analysis in the present study to be incorrect. Thus, although adult sex assessments made by experienced anthropologists were confirmed proteomically in the majority of cases, these findings demonstrate that proteomics approaches occupy an important and complementary role in anthropological research. This is particularly true in cases where the skeletal remains of adult individuals are poorly preserved, the pelvis is absent, and only fragmentary elements of other bones are available.

Proteomics sex estimation is particularly important in the case of nonadult individuals, for whom accurate and reliable sex assessment using morphological methods is not possible because the development of sexually dimorphic traits is incomplete. In this study, we analyzed 100 nonadult individuals from the Rajhrad site. Targeted MALDI-CASI-FTICR analysis identified 48 males and 51 females. The biological sex of one individual (H163) could not be determined by this method because the peptide signal was too weak to allow a potential female assignment. However, LC-HRMS analysis subsequently confirmed that this individual was female.

Although sex assessment is not routinely performed in nonadult individuals, sex was assessed for a total of 13 individuals in our sample by Pospíšilová^24^ using metric measurements of permanent teeth following the method proposed by Cardoso et al.^25^ Proteomics analysis confirmed the accuracy of these estimates in 11 of the 13 individuals, which can be considered a high success rate and indicates the good reliability of this method. Strong concordance between this metric approach and genetics sex estimation in nonadult individuals has also been demonstrated in a recent study^56^ and the method has been successfully applied in other contemporary research as well.^57^

## Conclusion

The newly developed high-throughput method for targeted paleoproteomics sex estimation using MALDI-CASI-FTICR mass spectrometry successfully estimated the biological sex of 129 to 130 adult and juvenile individuals from medieval Great Moravia. This approach combines the advantages of all methods commonly used to date, offering ultrahigh resolution, substantially reduced processing times, applicability to large archeological sample sets, and minimally invasive sampling. Internal quantification based on heavy/light peptide ratios further enhances the accuracy of biological sex estimation, ensuring reliability even in the presence of instrumental variations. The unique disadvantage of the targeted MALDI-CASI-FTICR method is the high cost of synthetic isotope-labeled peptides used as the internal standard compared to the synthetic peptides used as the external standard in targeted LC-HRMS.

The possibility of proteomics sex estimation in nonadult individuals opens up new opportunities for anthropologists and archeologists, including more accurate reconstructions of past population structures, improved analyses of mortality patterns and sex-specific burial practices, and the investigation of social organization, kinship, and gender-related behavior in past societies, which have previously been inaccessible due to the inability to reliably estimate sex in juvenile remains.

The method developed here still needs to be improved for the field of forensic medicine. The cohort should be expanded in order to refine specificity and false positive and negative rates. It is also important to have cohorts of individuals whose sex is known for different periods in order to determine the appropriate measures for each time period.

## Supplementary Material









## Data Availability

The mass spectrometry proteomics data have been deposited to the Zenodo Archive (https://zenodo.org/) via the Zenodo partner repository with the data set identifier 117817489 corresponding to the MALDI CASI FTICR data. LC-HRMS data have been deposited to the PRIDE partner repository^58^ with the data set identifier PXD08085.

## Notes

Supplementary figures (Figures S1–S16) and Table S1. The figures provide complementary MALDI-FTICR MS, MALDI-CASI-FTICR MS, and LC-HRMS data for modern and archaeological teeth, including peptide identification, AMELX/AMELY sequence coverage, sex estimation, peptide intensity ratios, and deamidation and peptide-length analyses. Table S1 lists peptides identified by MALDI-FTICR MS, including their sequences, post-translational modifications, corresponding proteins, molecular formulas, observed m/z values, and presence or absence in the spectra.SI1. Supporting Information containing supplementary figures (Figures S1–S16) and Table S1. The figures provide complementary MALDI-FTICR MS, MALDI-CASI-FTICR MS, and LC-HRMS data for modern and archaeological teeth, including peptide identification, AMELX/AMELY sequence coverage, sex estimation, peptide intensity ratios, and deamidation and peptide-length analyses. Table S1 lists peptides identified by MALDI-FTICR MS, including their sequences, post-translational modifications, corresponding proteins, molecular formulas, observed m/z values, and presence or absence in the spectra. (PDF)
Table S1, S2: Log2 intensities of AMELX, Y, Ratio 889/879, Ratio 1089/1079 and sex estimation for both MALDI-FTICR MS and MALDI-CASI-FTICR-MS for Rajhrad and Mikulčice site respectively (XLSX)
Identified peptides on juveniles, adults moderns samples and archeological samples by PEAKS­(XLSX)
Log2 AMELX and Log2 AMELY used in Figure 5 (XLSX)

## ○

Jaroslav Brůžek Deceased.
