# Supplementary material for: High-Throughput Targeted Paleoproteomics Sex Estimation on Medieval Great Moravian Individuals Using MALDI-CASI-FTICR Mass Spectrometry
Source: J Proteome Res. 2026 Aug 28;25(10):5173–88. doi: 10.1021/acs.jproteome.6c00163 (PMC13640426; doi:10.1021/acs.jproteome.6c00163)
Supplement: Supplementary file 1 [file pr6c00163_si_001.pdf]

High-throughput targeted paleoproteomics sex estimation on medieval Great Moravia individuals using MALDI-CASI-FTICR mass spectrometry

Fabrice Bray[1,§], Anežka Pilmann Kotěrová[2,§], Lisa Garbé[1], Marc Haegelin[1], Benoît Bertrand[3], Kevimy Agossa[4], Christian Rolando[1], Petr Velemínský[5], Jaroslav Brůžek[2,‡,†], Marine Morvan[6,‡,*]

[1] Univ. Lille, CNRS, UAR 2638 - IMEC – Institut Michel Eugène Chevreul, F-59000 Lille, France

[2] Department of Anthropology and Human Genetics, Faculty of Science, Charles University, Viničná 7, 128 00 Prague 2, Czech Republic

[3] Univ. Lille, CHU Lille, ULR 7367 - UTML&A - Unité de Taphonomie Médico-Légale & d'Anatomie, F-59000 Lille, France

[4] Univ. Lille, CHU Lille, School of Dentistry, Department of Periodontology, 59000 Lille, France

[5] Department of Anthropology, National Museum, Václavské náměstí 1700/68, 110 00 Prague 1, Czech Republic

[6] Vilnius University, Faculty of History, Department of Archaeology, Universiteto g. 7, LT-01513 Vilnius, Lithuania

§ These authors contributed equally to this work

‡ These authors jointly directed this study

[†] Deceased

[*] marine.morvan@palaeoproteomics.org (MM)

Figure S 1: MS spectra of modern male and female reference analyzed by MALDI -FTICR MS. 3
Figure S 2: Zoom in the region m/z 800 to 1,100 of the spectra of modern male and female reference analyzed by MALDI -FTICR MS. Peptides form AMELX, Y, Ameloblastin and COL1A1 were annotated. .3
Figure S 3: Zoom in the region m/z 861 to 868 of the spectra of modern male reference analyzed by MALDI -FTICR MS. Peptide M(oxidized)PLPPHPG and SMIRPPY are indicated. A zoom was taken of the peak corresponding to the second isotope of the M(oxidized)PLPPHPG peptide and the monoisotopic peak of the SMIRPPY peptide. 4
Figure S 4: Sequence coverage of AMELX and AMELY identified for one male and female adult modern by LC-HRMS. The result was obtained with PEAKS 13. The blue bar corresponding to the matching peptides. d blue = deamidation, p purple = phosphorylation, o turquoise = oxidation 8
Figure S 5: Sequence coverage of AMELX and AMELY identified for male and female juvenile modern by LC-HRMS. The result was obtained with PEAKS 13. The blue bar corresponding to the matching peptides. d blue = deamidation, p purple = phosphorylation, o turquoise = oxidation 8
Figure S 6: MS spectra of modern male reference analyzed by MALDI-CASI-FTICR (up) and MALDI-FTICR MS (down). A) Full MS spectra and B) zoom in on the spectrum for the mass range *m/z* 815-1,115.....9
Figure S 7: Overview of sex estimation of the 28 modern teeth by MALDI-CASI-FTICR, represented as the Log_2 AMELY intensity as a function of Log_2 AMELX1 intensity (A) or AMELX2L (B) for each sample. The blue and green dots correspond to males and females, respectively. 10
Figure S 8: Log_2 ratio of intensities for the peptide *m/z* 889.4491 / *m/z* 879.4393 and *m/z* 1079.5520 / *m/z* 1089.5621 for 28 adult modern teeth...11
Figure S 9: Overview of biological sex estimation for archaeological samples by MALDI-CASI-FTICR, represented as the Log_2 AMELY intensity as a function of Log_2 AMELX1 intensity. Biological sex estimation of the Mikulčice (A) and Rajhrad (B) sites. Blue dots correspond to males, green dots to females, and orange dots correspond to indeterminate samples. 11
Figure S 10: Log_2 ratio of intensities for the peptide *m/z* 889.4491 / *m/z* 879.4393 and *m/z* 1079.5520 / *m/z* 1089.5621 for (A) Mikulčice site and (B) Rajhrad site...12
Figure S 11: Sequence coverage of AMELX and Y form H163 sample. The result was obtained with PEAKS 13. The blue bar corresponding to the matching peptides. d blue = deamidation, p purple = phosphorylation, o turquoise = oxidation 12
Figure S 12: Box plot of deamidation on Q (glutamine) left panel and N (asparagine) right panel on AMELX for modern and archaeological teeth analyzed by LC-HRMS. 12
Figure S 13: Box plot of deamidation on Q (glutamine) left panel and N (asparagine) right panel on AMELY for modern and archaeological teeth analyzed by LC-HRMS. 13
Figure S 14: Box plot of deamidation on Q (glutamine) left panel and N (asparagine) right panel on COL1A1 for modern and archaeological teeth analyzed by LC-HRMS. 13
Figure S 15: Box plot of deamidation on Q (glutamine) left panel and N (asparagine) right panel on COLA1A2 for modern and archaeological teeth analyzed by LC-HRMS. 13
Figure S 16: Box plot of length of peptide identified on proteins for modern and archaeological teeth analyzed by LC-HRMS. 14

Table S 1: Peptides identified in MALDI FTICR spectra in modern and juvenile. The table contains the sequence of peptide with PTMs, the proteins, the chemical formula, $[M+H]^+$ observed and the presence "v" or absence "x" in the spectra. 5

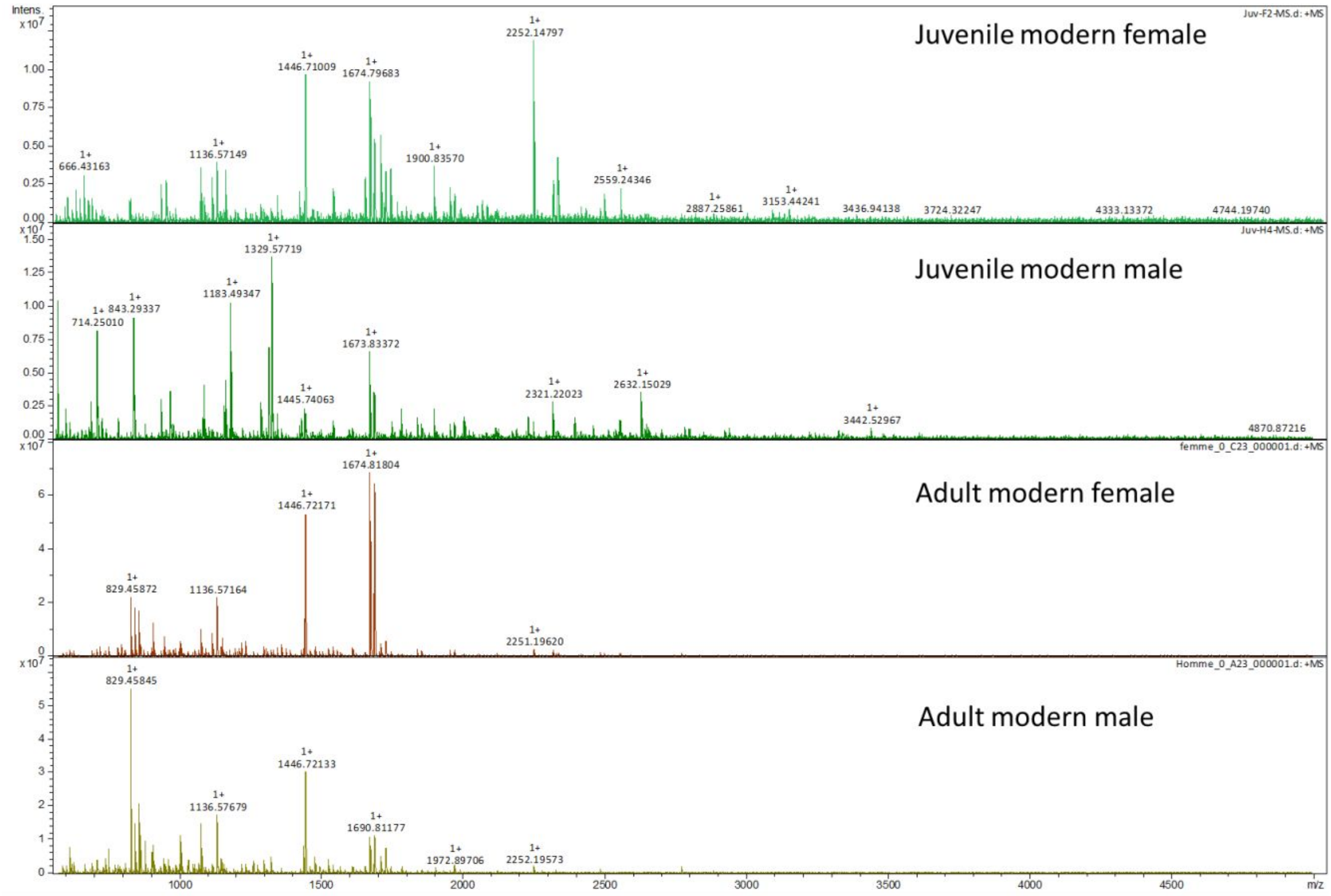

Figure S 1: MS spectra of modern male and female reference analyzed by MALDI -FTICR MS.

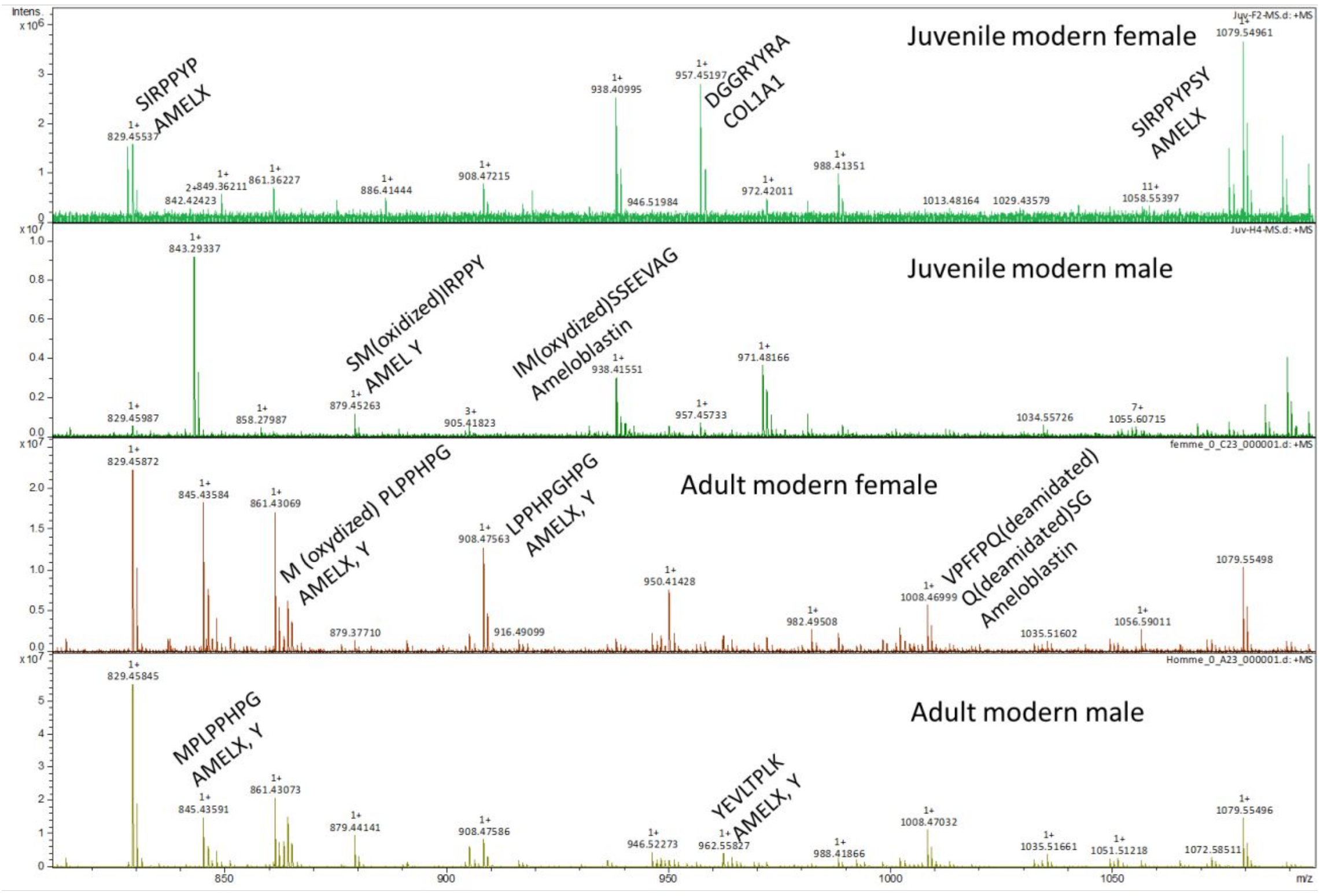

Figure S 2: Zoom in the region *m/z* 800 to 1,100 of the spectra of modern male and female reference analyzed by MALDI -FTICR MS. Peptides form AMELX, Y, Ameloblastin and COL1A1 were annotated.

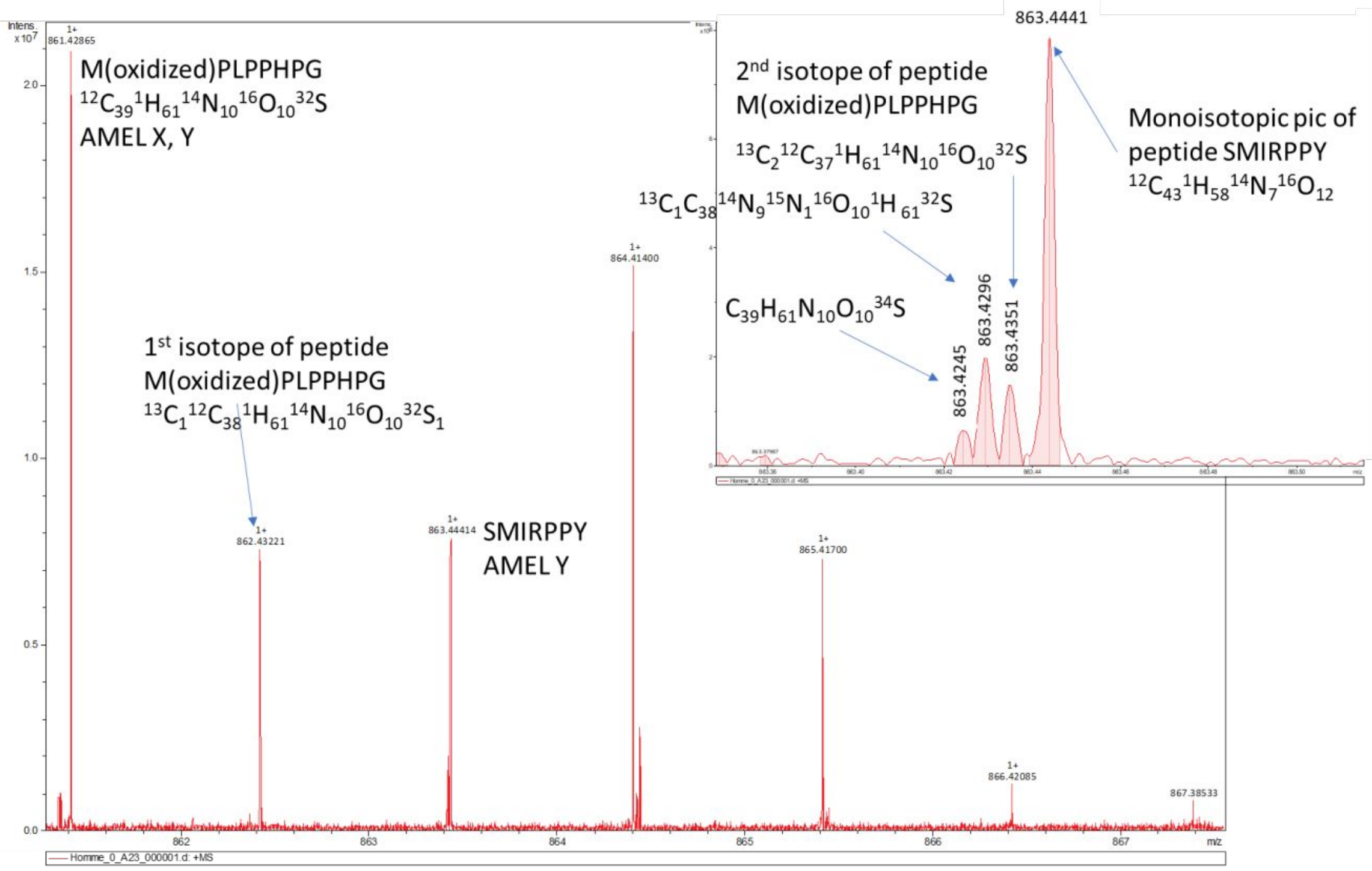

Figure S 3: Zoom in the region *m/z* 861 to 868 of the spectra of modern male reference analyzed by MALDI -FTICR MS. Peptide M(oxidized)PLPPHPG and SMIRPPY are indicated. A zoom was taken of the peak corresponding to the second isotope of the M(oxidized)PLPPHPG peptide and the monoisotopic peak of the SMIRPPY peptide.

Table S 1: Peptides identified in MALDI FTICR spectra in modern and juvenile. The table contains the sequence of peptide with PTMs, the proteins, the chemical formula, $[M+H]^+$ observed and the presence “v” or absence “x” in the spectra.

				**Modern**	**Modern**	**Archaeology**	**Archaeology**
**Peptide Sequence**	**Proteins**	**Formula**	**$[M+H]^+$**	**Juvenile**	**Adults**	**Juvenile**	**Adults**
LPPHPG	XY	C29H45N8O7	617.3405	x	v	v	v
IRPPYP	X	C36H56N9O8	742.4246	x	v	v	v
SIRPPYP	X	C39H61N10O10	829.4566	v	v	v	v
MPLPPHPG	XY	C39H61N10O9S	845.4338	v	v	v	v
M(oxidized)PLPPHPG	XY	C39H61N10O10S	861.4287	v	v	v	v
SMIRPPY	Y	C39H63N10O10S	863.4443	x	v	v	v
SM(oxidized)IRPPY	Y	C39H61N10O11S	879.4393	v	v	v	v
TDKTKQEEVD	XY	C49H86N15O21	1192.5691	x	v	v	v
TDKTKQ(deamidated)EEVD	XY	C48H81N12O23	1193.5532	v	v	v	v
STDKTKREEVD	XY	C52H91N16O23	1307.6437	v	v	v	v
LPPHPGHPG	XY	C42H62N13O10	908.4737	x	x	v	v
YEVLTPLK	XY	C46H76N9O13	962.5557	x	v	v	v
SYEVLTPLK	XY	C49H81N10O15	1049.5877	x	v	v	v
MPLPPHPGHP	XY	C50H75N14O11S	1079.5454	x	v	v	v
MPLPPHPGHPG	XY	C52H78N15O12S	1136.5669	v	v	v	v

M(oxidized)PLPPHPGHPG	XY	C52H78N15O13S	1152.5618	x	v	v	v
FS(phosphorylation)YEVLTPLK	X	C58H91N11O19P	1276.6224	x	v	v	v
LPPHPGHPGYINF	XY	C70H97N18O16	1445.7324	v	v	v	v
LPPHPGHPGYIN(deamidated)F	XY	C70H96N17O17	1446.7164	x	v	v	v
SIRPPYPSYGYEPM(oxidized)	XY	C77H110N17O23S	1672.7675	v	v	v	v
MPLPPHPGHPGYINF	XY	C80H113N20O18S	1673.8256	x	v	v	v
M(oxidized)PLPPHPGHPGYINF	XY	C80H113N20O19S	1689.8206	x	v	v	v
M(oxidized)PLPPHPGHPGYIN(deamidated)F	XY	C80H112N19O20S	1690.8046	x	v	v	v
M(oxidized)PLPPHPGHPGYINFS(deamidated)	XY	C83H118N21O24PS	1855.8111	v	v	v	v
SEEVAGGREDPM(oxidized)A	Ameloblastin	C53H87N16O24S	1363.5794	v	v	v	v
SLQ(deamidated)RLN(deamidated)TLSQ(deamidated)Y	Ameloblastin	C57H92N14O22	1325.6582	x	v	v	v
VPFFPQ(deamidated)Q(deamidated)SG	Ameloblastin	C48H65N9O15	1008.4672	x	v	v	v
VPFFPQ(deamidated)Q(deamidated)SGTPGM(oxidized)A	Ameloblastin	C67H96N14O22S1	1481.6616	x	v	v	v
VPFFPQ(deamidated)Q(deamidated)	Ameloblastin	C43H57N7O12	864.1377	x	v	v	v
VPFFPQ(deamidated)Q(deamidated)SGTPG	Ameloblastin	C59H82N12O19	1263.5891	v	v	v	v
FGGRPPYY	Enamelin	C47H62N11O11	956.4624	v	v	v	v
LPPPGYGRPPIS(phosphorylation)NEEGG	Enamelin	C77H119N21O28P	1816.8265	x	v	v	v
GFGGRPPYY	Enamelin	C49H65N12O12	1013.4839	x	v	v	v

LPPPGYGRPPIS(phosphorylation)NEEGGNPYFG	Enamelin	C106H153N27O35P	2395.0754	v	v	v	v
GRPPYY	Enamelin	C36H50N9O9	752.3726	x	v	v	v
GRPPIS(phosphorylation)NEEG	Enamelin	C43H72N14O20P	1135.4779	v	v	v	v
DGGRYYRA	COL1A1	C41H61N14O13	957.4537	v	v	v	v
IM(oxydized)SSEEVAG	Ameloblastin	C37H64N9O17S	938.4135	v	v	v	v
LDGAKGDAGPA	COL1A1	C40H67N12O16	971.4792	v	v	x	x
VPFFPQ(deamidated)Q(deamidated)SG	Ameloblastin	C48H66N9O15	1008.4672	x	v	v	v

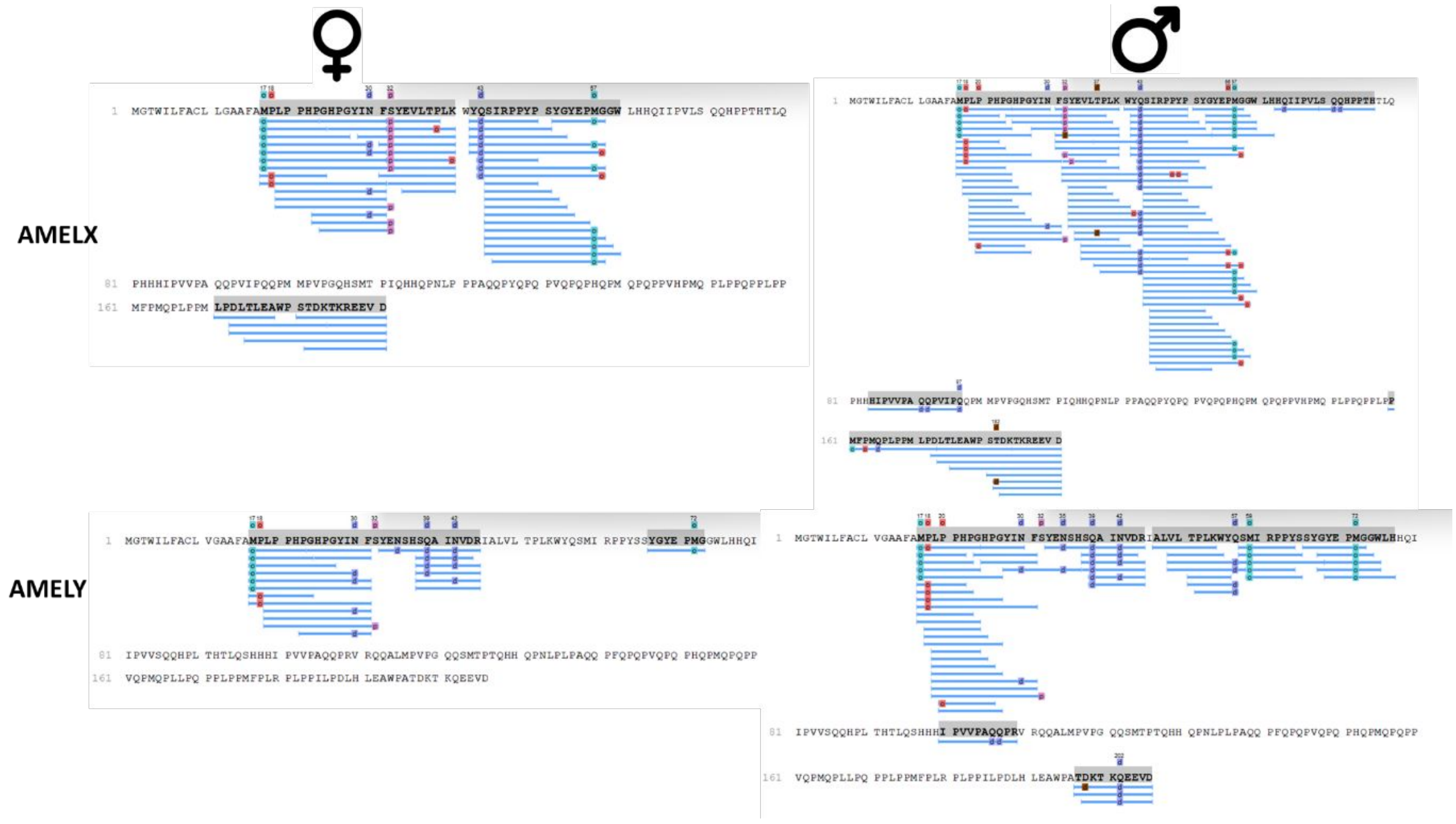

Figure S 4: Sequence coverage of AMELX and AMELY identified for one male and female adult modern by LC-HRMS. The result was obtained with PEAKS 13. The blue bar corresponding to the matching peptides. d blue = deamidation, p purple = phosphorylation, o turquoise = oxidation

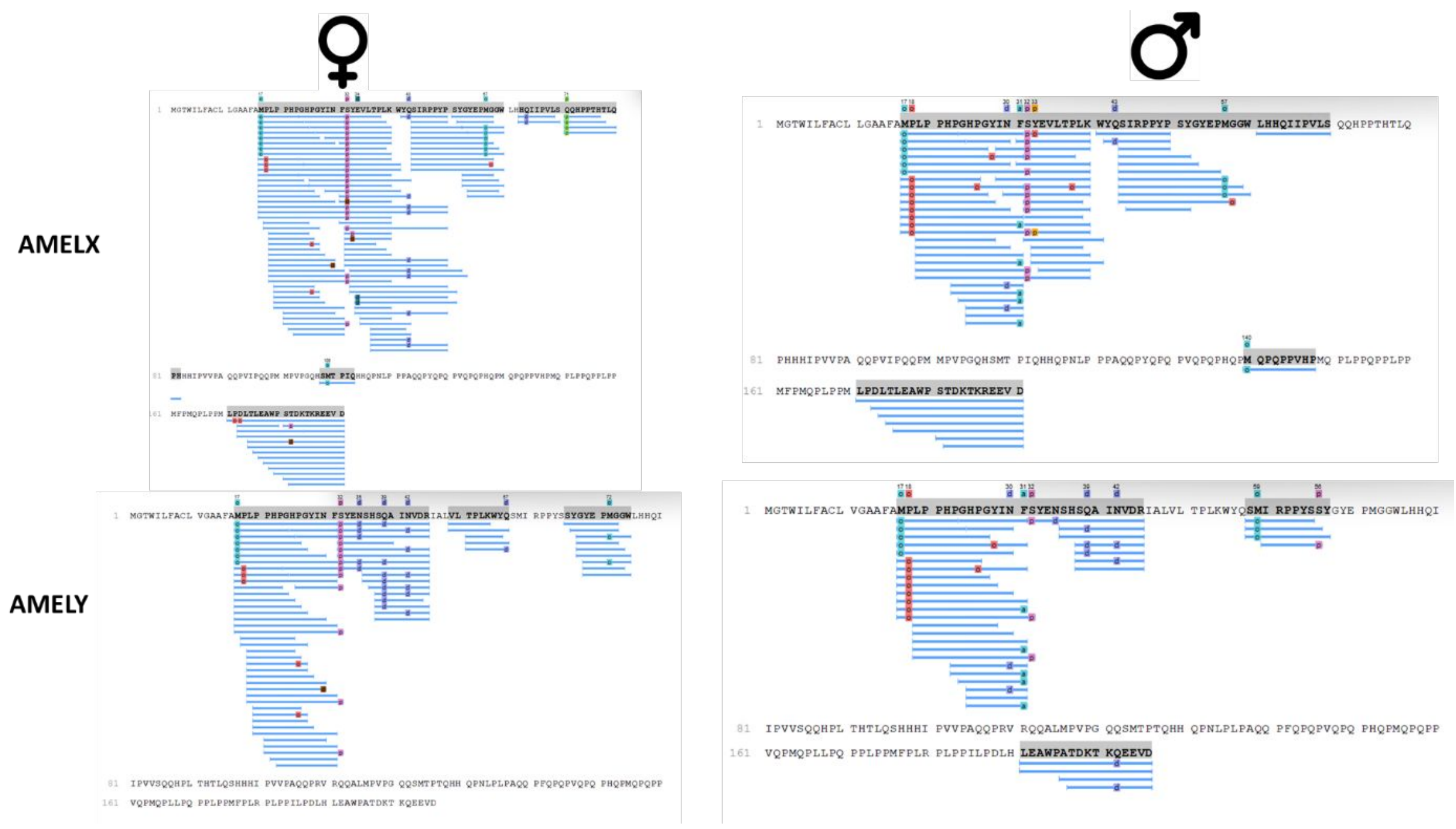

Figure S 5: Sequence coverage of AMELX and AMELY identified for male and female juvenile modern by LC-HRMS. The result was obtained with PEAKS 13. The blue bar corresponding to the matching peptides. d blue = deamidation, p purple = phosphorylation, o turquoise = oxidation

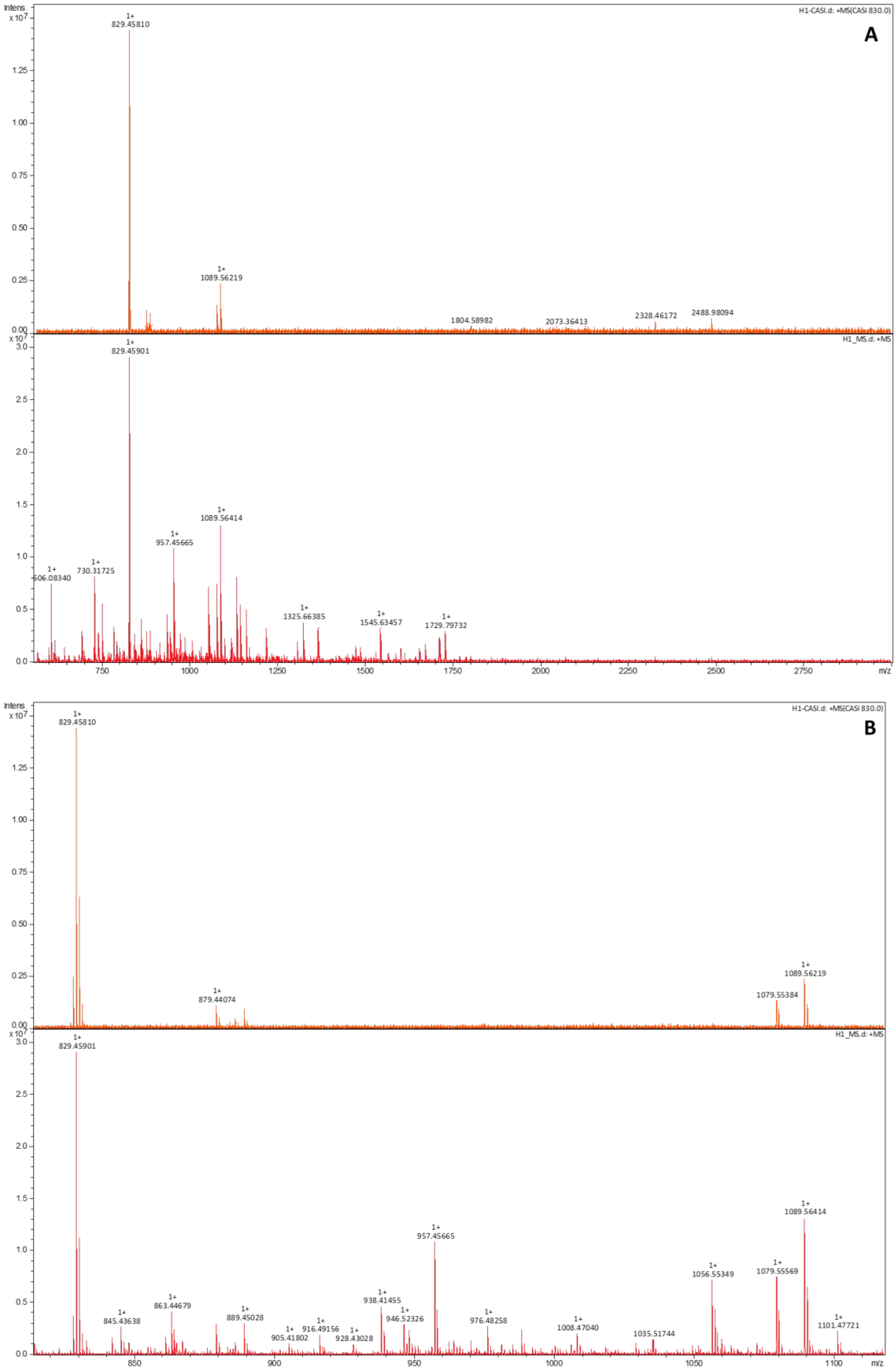

Figure S 6: MS spectra of modern male reference analyzed by MALDI-CASI-FTICR (up) and MALDI-FTICR MS (down). A) Full MS spectra and B) zoom in on the spectrum for the mass range *m/z* 815-1,115.

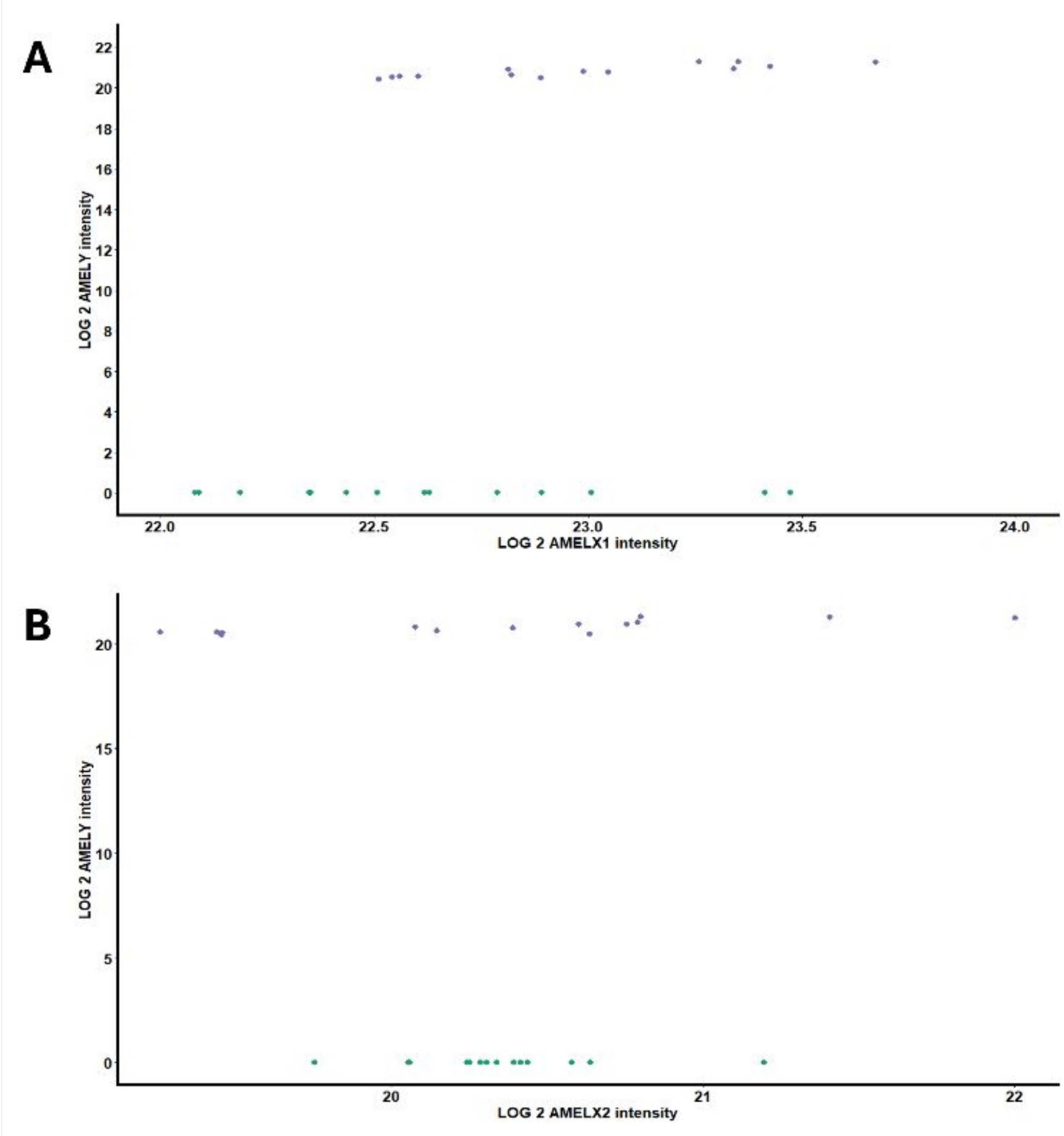

Figure S 7: Overview of sex estimation of the 28 modern teeth by MALDI-CASI-FTICR, represented as the Log_2 AMELY intensity as a function of Log_2 AMELX1 intensity (A) or AMELX2L (B) for each sample. The blue and green dots correspond to males and females, respectively.

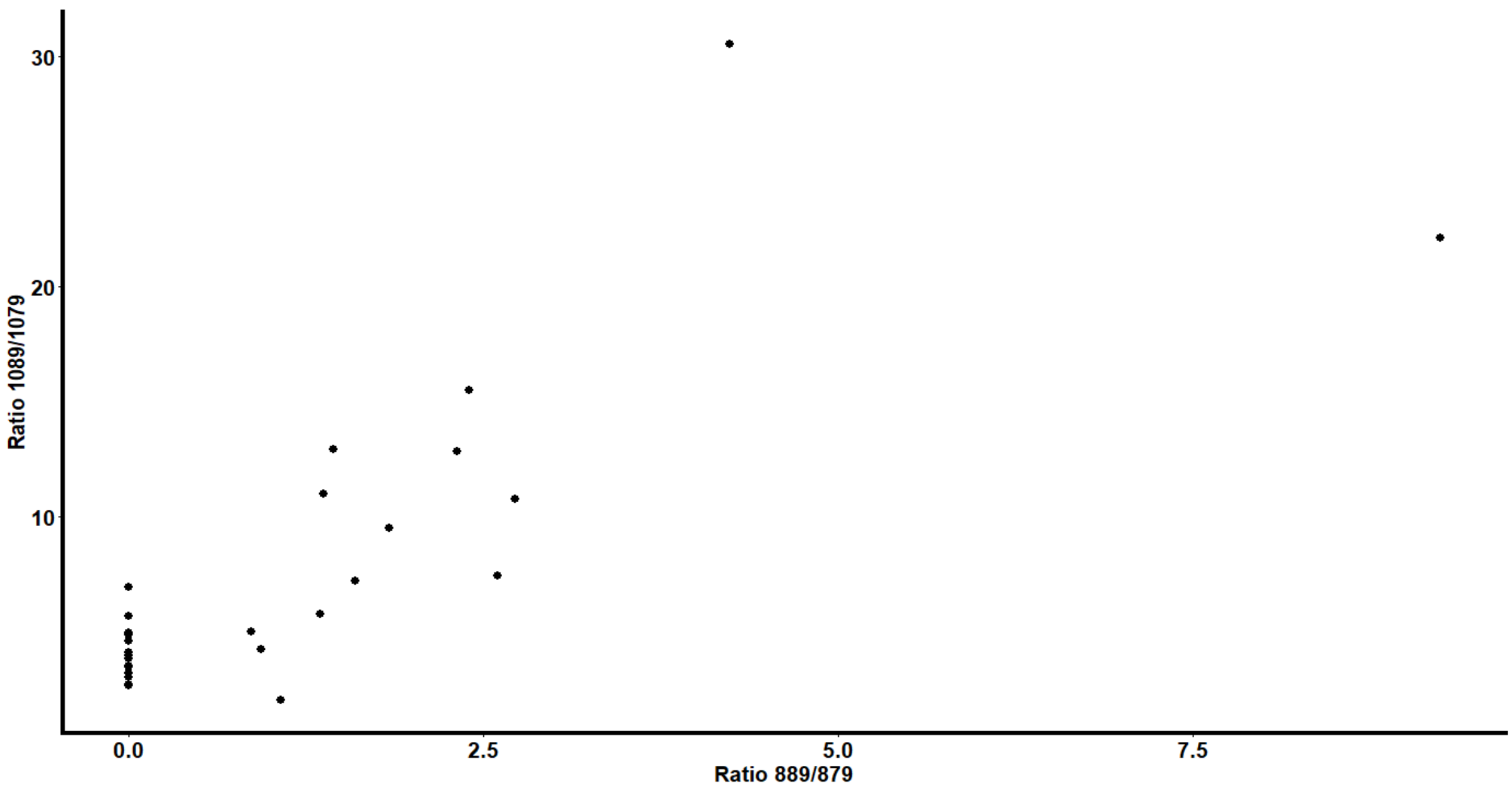

Figure S 8: Log_2 ratio of intensities for the peptide *m/z* 889.4491 / *m/z* 879.4393 and *m/z* 1079.5520 / *m/z* 1089.5621 for 28 adult modern teeth.

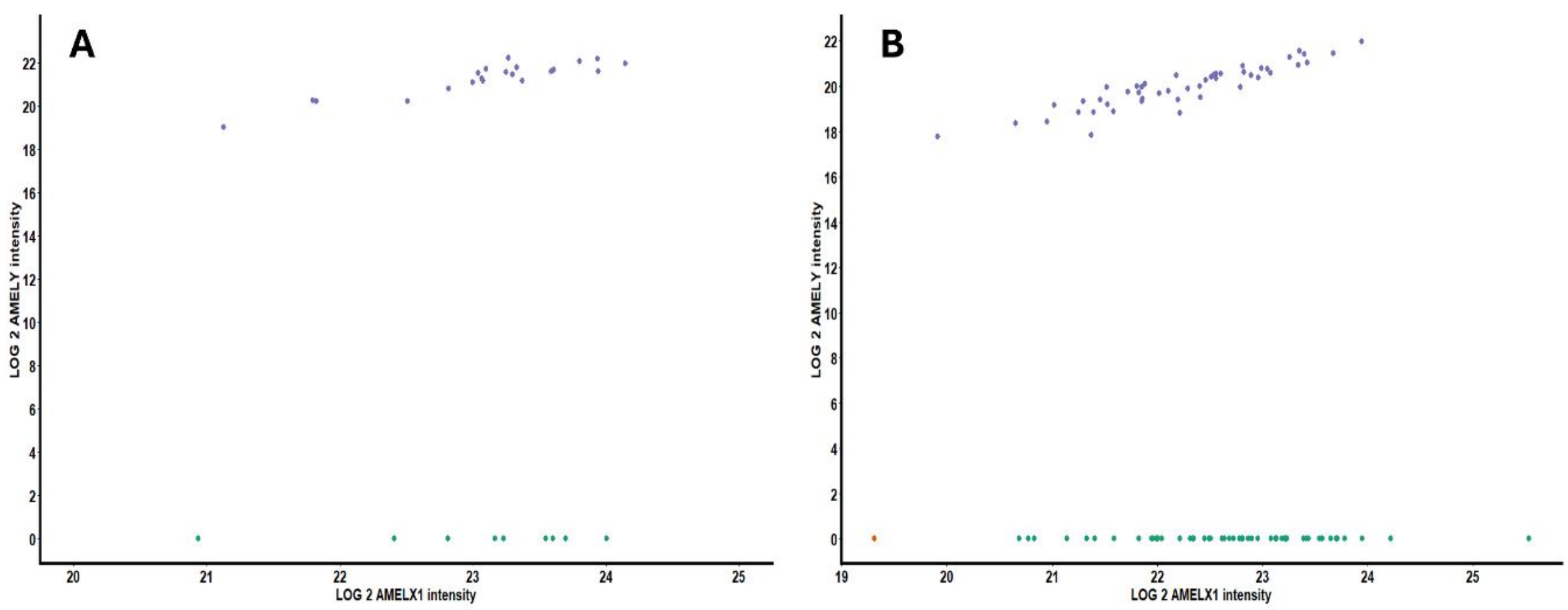

Figure S 9: Overview of biological sex estimation for archaeological samples by MALDI-CASI-FTICR, represented as the Log_2 AMELY intensity as a function of Log_2 AMELX1 intensity. Biological sex estimation of the Mikulčice (A) and Rajhrad (B) sites. Blue dots correspond to males, green dots to females, and orange dots correspond to indeterminate samples.

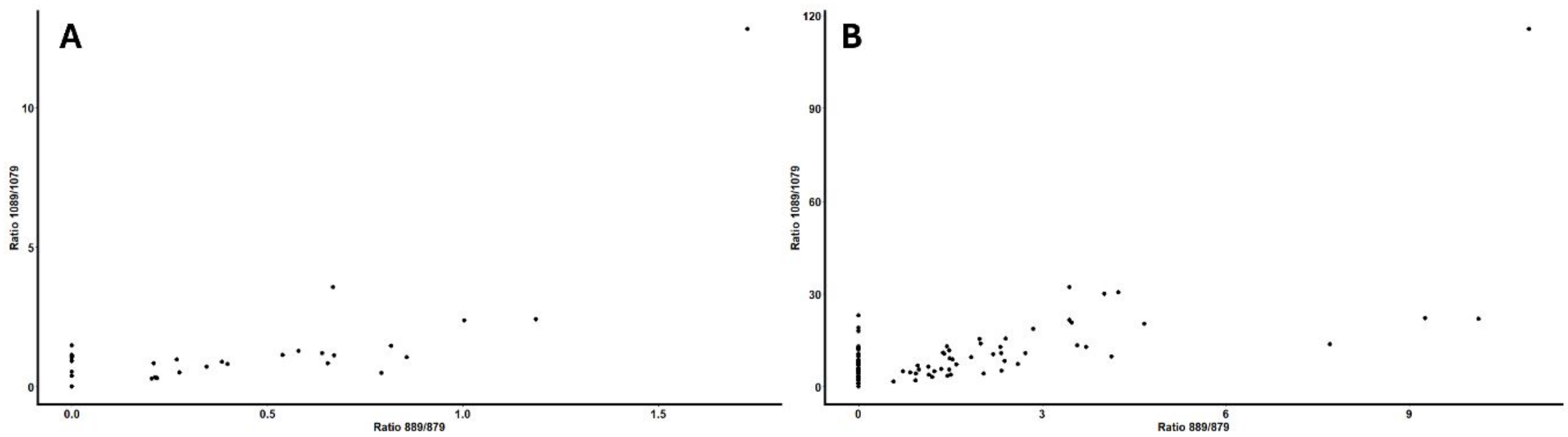

Figure S 10: Log_2 ratio of intensities for the peptide *m/z* 889.4491 / *m/z* 879.4393 and *m/z* 1079.5520 / *m/z* 1089.5621 for (A) Mikulčice site and (B) Rajhrad site.

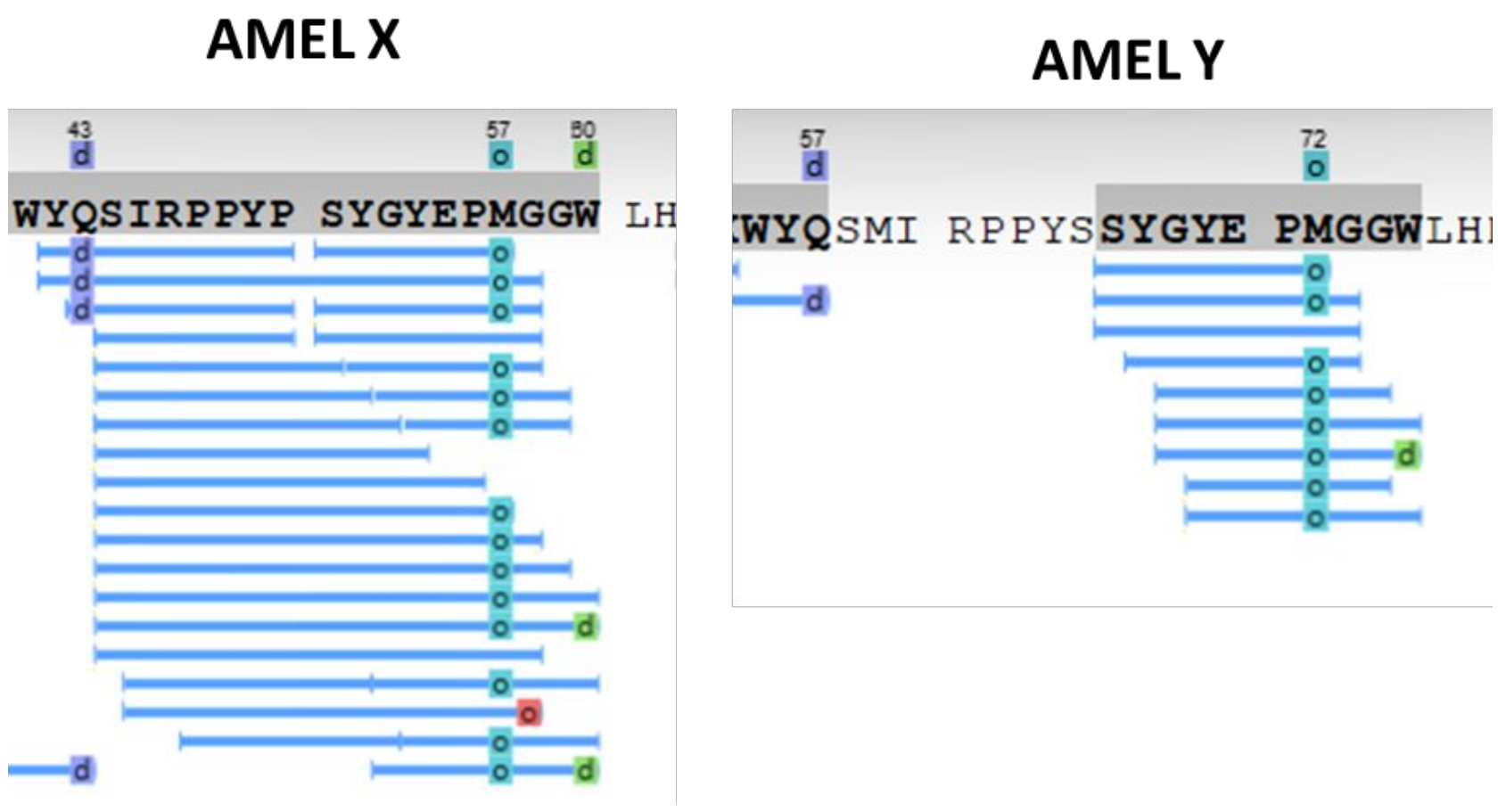

Figure S 11: Sequence coverage of AMELX and Y form H163 sample. The result was obtained with PEAKS 13. The blue bar corresponding to the matching peptides. d blue = deamidation, p purple = phosphorylation, o turquoise = oxidation

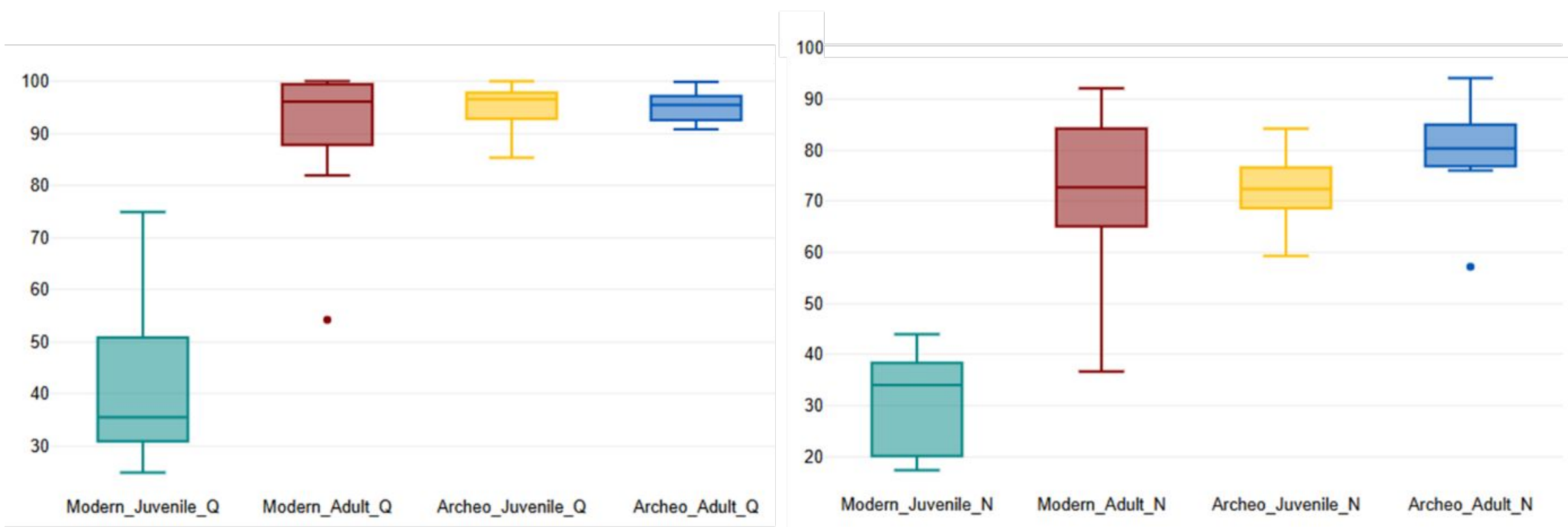

Figure S 12: Box plot of deamidation on Q (glutamine) left panel and N (asparagine) right panel on AMELX for modern and archaeological teeth analyzed by LC-HRMS.

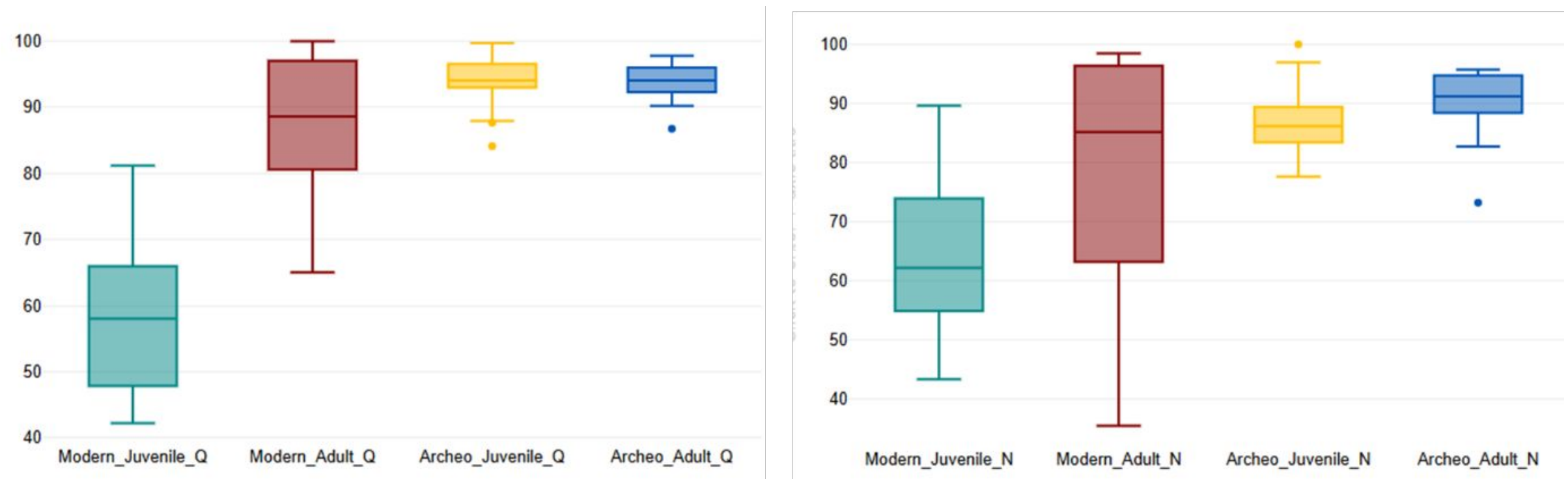

Figure S 13: Box plot of deamidation on Q (glutamine) left panel and N (asparagine) right panel on AMELY for modern and archaeological teeth analyzed by LC-HRMS.

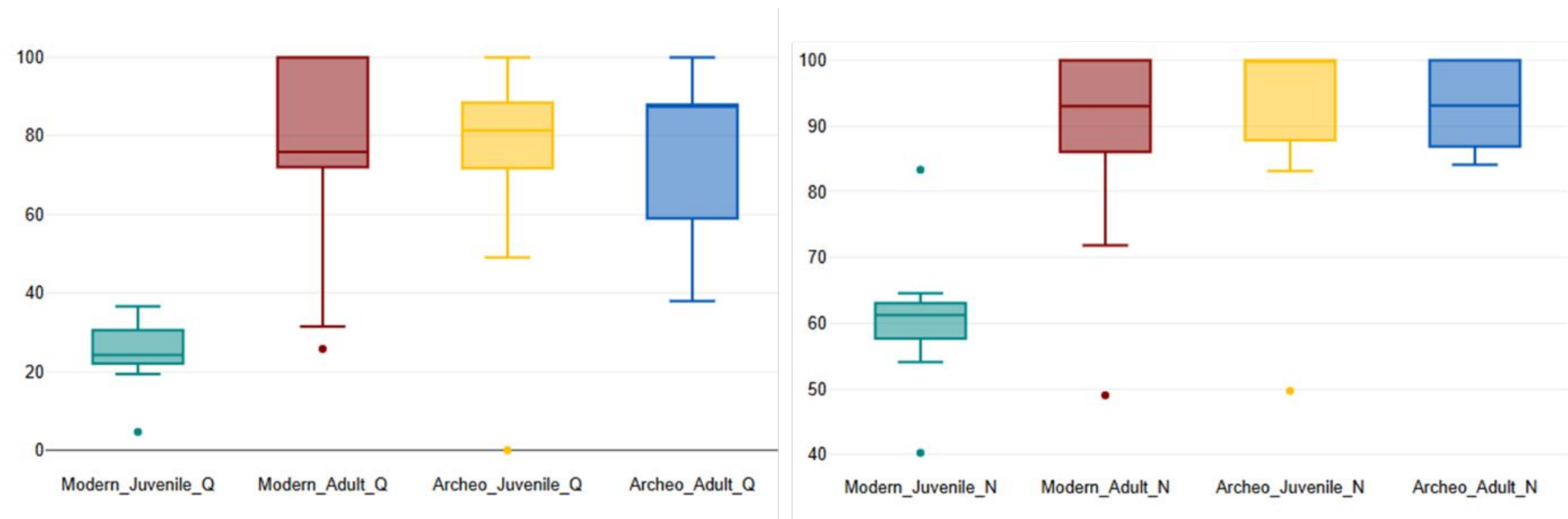

Figure S 14: Box plot of deamidation on Q (glutamine) left panel and N (asparagine) right panel on COL1A1 for modern and archaeological teeth analyzed by LC-HRMS.

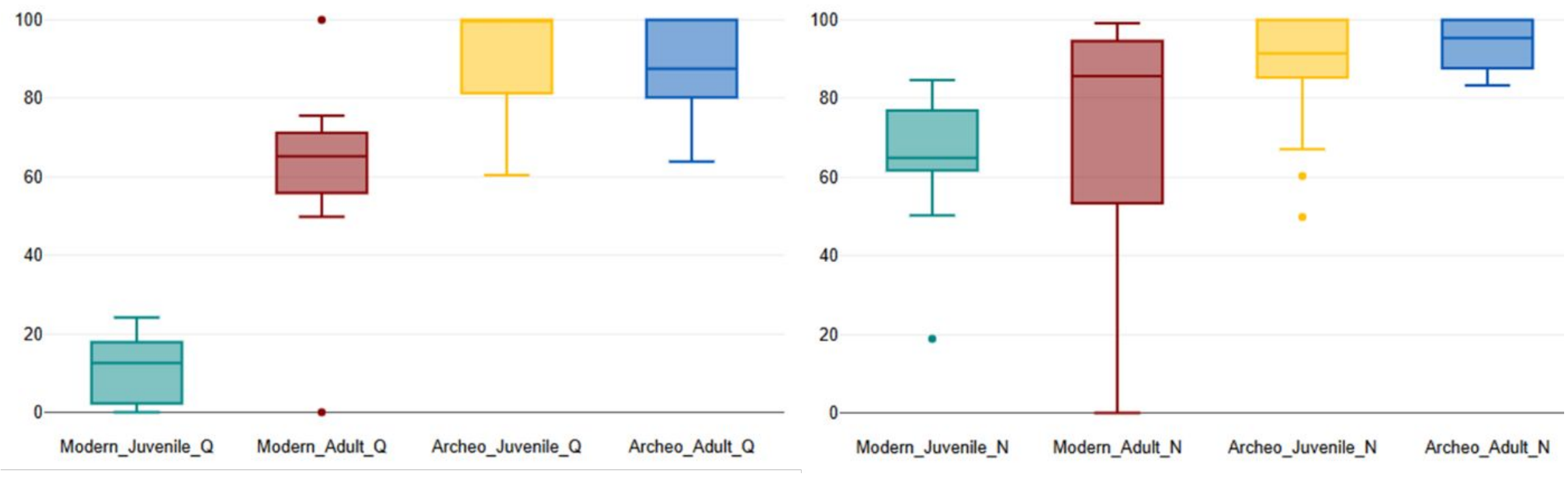

Figure S 15: Box plot of deamidation on Q (glutamine) left panel and N (asparagine) right panel on COLA1A2 for modern and archaeological teeth analyzed by LC-HRMS.

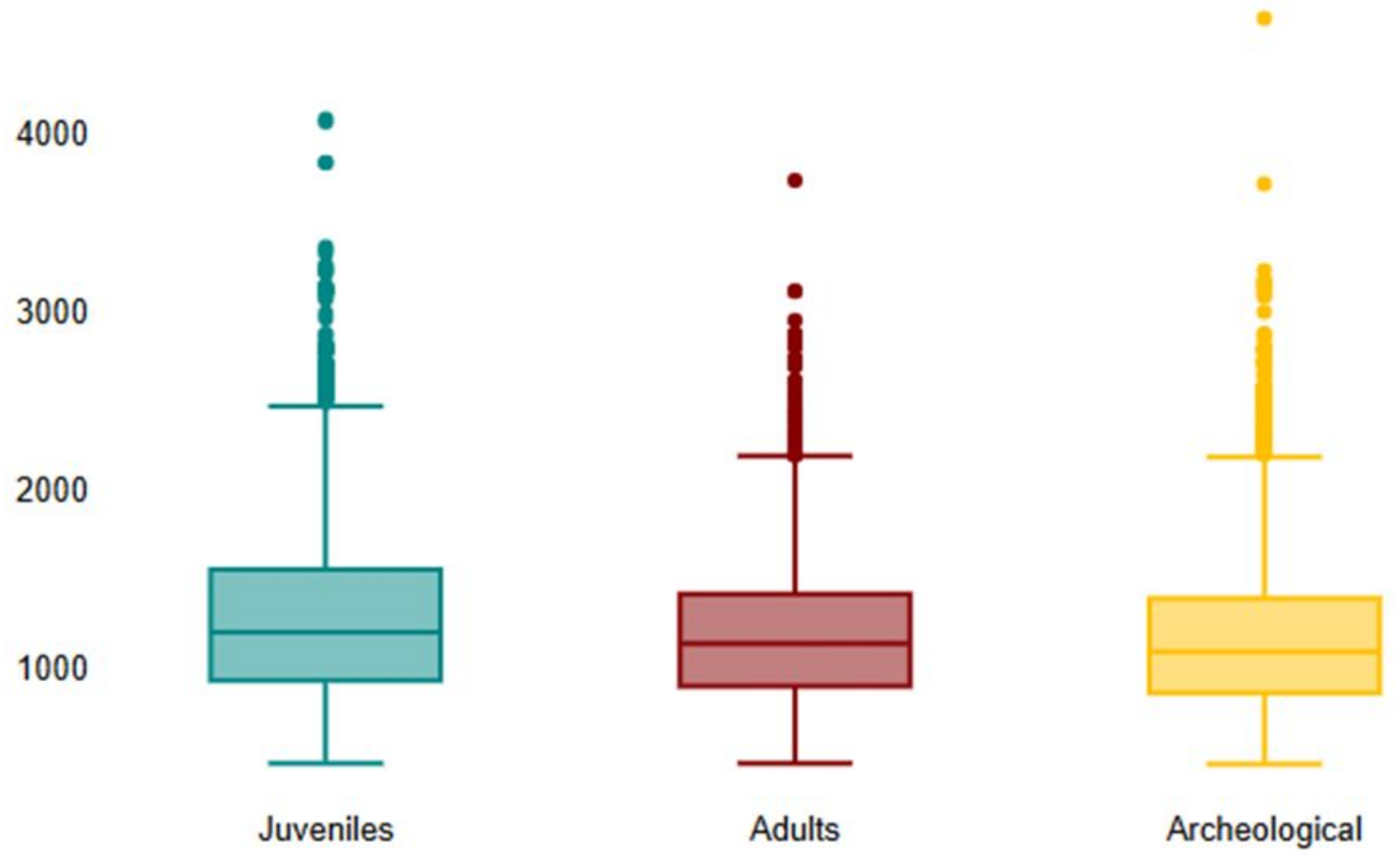

Figure S 16: Box plot of length of peptide identified on proteins for modern and archaeological teeth analyzed by LC-HRMS.
